# Electrically pumped surface-emitting amplified spontaneous emission from colloidal quantum dots

**DOI:** 10.1038/s41377-025-01972-1

**Published:** 2025-08-19

**Authors:** Fengshou Tian, Tianhong Zhou, Xuanyu Zhang, Rui Chen, Shuming Chen

**Affiliations:** State Key Laboratory of Quantum Functional Materials, Department of Electrical and Electronic Engineering, Southern University of Science and Technology, Shenzhen, 518055 China

**Keywords:** Inorganic LEDs, Quantum dots

## Abstract

Colloidal quantum dots (QDs) are promising gain materials for realizing solution-processable, wavelength-tunable and low-cost laser diodes. However, achieving electrically pumped amplified spontaneous emission (ASE) in QDs, a prerequisite for lasing, is hampered by the low net optical gain and low current injection of the diodes. Here we demonstrate electrically pumped and surface-emitting ASE from QDs by electro-thermal-optically co-designing a quantum-dot light-emitting diode (QLED) with high net optical gain and high current injection. By developing a top-emitting cavity featuring a Ag/indium-zinc-oxide (IZO) bottom reflective electrode and a IZO/Ag top semi-transparent electrode, the QD emission is effectively resonated; moreover, not only are the surface plasmon polariton losses induced by the metallic electrodes completely eliminated, but also the optical field can be confined primarily within the QDs, resulting in a reduction in loss and a 2-fold enhancement in gain. As a result, the QLED exhibits surface-emitting ASE with a threshold of 10 μJ cm^−2^ when pumped by a 100 fs laser at 77 K. By building the QLED directly on a Si heat sink and driving the QLED with an ns-pulsed current source, the Joule heat is effectively dissipated, allowing the QLED to operate stably even at a high current of 2000 A cm^−2^. At 153 K and an injection current of 94 A cm^−2^, the QLED demonstrates surface-emitting ASE with strong directionality, high intensity and narrow bandwidth. The developed QLED, capable of generating surface-emitting ASE, paves the way for the development of QD based vertical cavity surface-emitting laser diodes.

## Abstract (web summary)

Electrically pumped and surface-emitting ASE from solution-casted colloidal QDs has been demonstrated by electro-thermal-optically co-designing of a QLED with high optical gain and large current injection.

## Graphical abstract

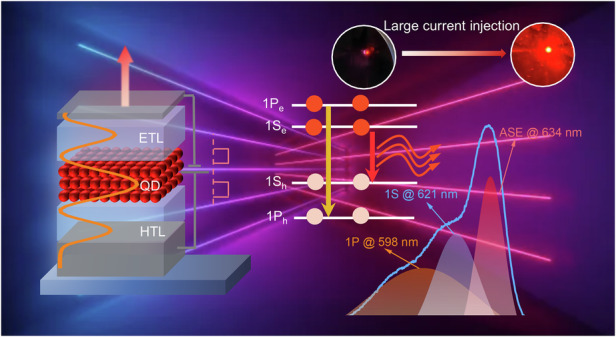

## Introduction

Laser diodes based on solution-processable gain materials such as organic molecules^1,2^, perovskites^3–5^, and colloidal quantum dots (QDs)^6–11^ have long been pursued due to their advantages of simple fabrication, low cost and high compatibility with various substrates, making them easy to integrate with on-chip photonics and electronics^12,13^. Among these gain materials, QDs, uniquely featuring size-tunable emission color, high photoluminescence (PL) quantum yield and high thermal stability^14–16^, are the most promising gain media for solution-processed lasers^17,18^. Optically pumped amplified spontaneous emission (ASE) and lasing from QD thin films have been demonstrated with a low threshold of 6–14^6,19^ and 17 μJ cm^−2 ^^19^, respectively, inspiring the exploration of electrically pumped ASE and even lasing from QDs. Recently, the successful demonstration of optically pumped surface-emitting lasing from an operational quantum-dot light-emitting diode (QLED)^9,10^ suggests a high possibility of realizing QD laser diodes.

However, electrically pumped surface-emitting ASE from QDs, a prerequisite for lasing, has not yet been demonstrated. To initiate ASE, the QDs should be pumped to achieve a population inversion, and to lower the ASE threshold, the QDs should be placed in a resonant cavity with high net modal gain. These require the electro-thermal-optical co-design of a cavity QLED with both high current injection and low optical losses, which remains a critical unsolved challenge. For example, the minimum current required for population inversion typically ranges from 3 to 4 A cm^−2 ^^8^, which can generate a large amount of Joule heat, posing a significant challenge to the stable operation of QLED. With effective thermal management such as reducing the emission area^8^, using a heat sink substrate^20^, driving the device with ns-pulsed current^5,21^, and operating the device at low temperature^5^, recent efforts have realized QLEDs with high injection current up to 1000 A cm^−2 ^^8^. At such a high injection current, each QD is occupied by multiple excitons, resulting in complete population inversion of both the band-edge (1S) and the higher energy (1P) transition^8^. However, ASE is still not achievable due to the high optical losses of the QLEDs, mainly caused by nonradiative recombination of the excitons, high absorption of the charge-conducting layers and poor field confinement in the QD gain medium. Very recently, Klimov’s group designed a cavity consisting of a distributed Bragg reflector (DBR) and a silver (Ag) top electrode, which improves field confinement in the QD gain medium and thus enables the realization of electrically pumped edge-emitting ASE^11^. However, surface-emitting ASE is more desirable, because the resulting vertical cavity surface-emitting lasers (VCSEL) are superior to edge-emitting lasers in terms of high beam quality, compact structure, simple packaging, ease of array integration and fiber coupling^22^. The realization of surface-emitting ASE is more difficult due to the short light propagation (amplification) path in the QD gain medium. Moreover, the surface-emitting structures require a semi-transparent electrode for light escaping, which reduces the optical field confinement in the QDs and increases the optical losses. Therefore, electrically pumped surface-emitting ASE from QDs has not yet been demonstrated.

Here, we address the above critical challenges by electro-thermal-optically co-designing a QLED that simultaneously possess high current injection and high net optical gain, enabling us to achieve electrically pumped and surface-emitting ASE. Firstly, by effective thermal management, we achieve a QLED with an injection current up to 2000 A cm^−2^, which is sufficiently high to induce a population inversion in QDs and leads to strong emission from both 1S and 1P transitions. Secondly, we design a top-emitting (TE) Fabry–Pérot (FP) cavity featuring an Ag/indium-zinc-oxide (IZO) bottom reflective electrode and an IZO/Ag top semi-transparent electrode, which effectively resonates the QD emission and lowers the ASE threshold. Thirdly, we introduce the dual IZO phase tuning layers, which, on the one hand, completely eliminate the surface plasmon polariton (SPP) losses induced by the Ag electrodes and, on the other hand, strongly confine the optical field in the QD gain medium, resulting in a 2-fold enhancement in net gain. As a result, surface-emitting ASE is achieved with a threshold of 10 μJ cm^−2^ (optically pumped) or 94 A cm^−2^ (electrically pumped). The developed QLED, capable of generating surface-emitting ASE, represents a promising device platform for realizing VCSEL diodes.

## Results and discussion

### Spectroscopic properties of QD gain medium

**Fig. 1:**
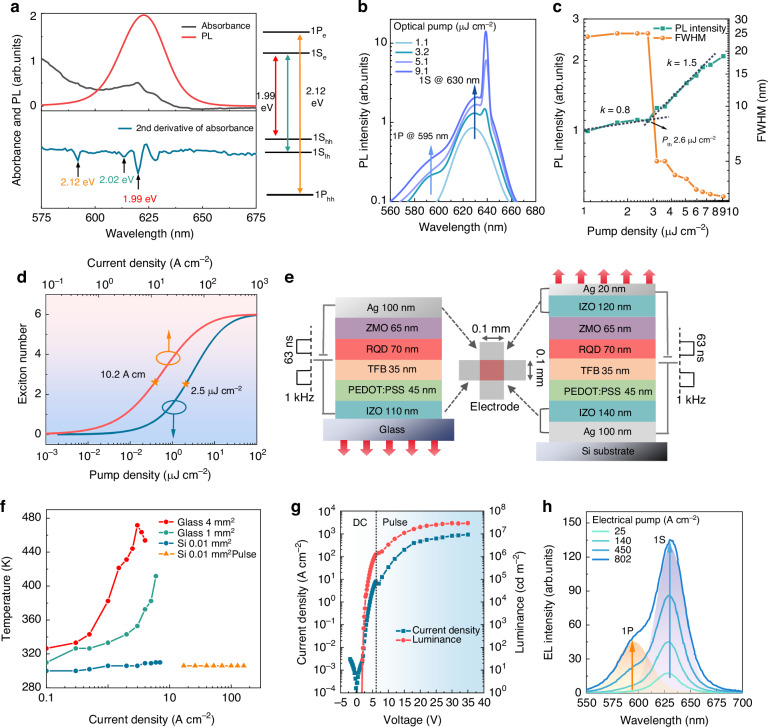
**Spectroscopic properties of QD gain medium and realization of high current injection QLEDs for population inversion**. **a** The PL (red), absorption (black) and absorption second derivative (blue) spectra of the QDs. The arrows mark the 1S_e_−1S_hh_, 1S_e_−1S_lh_ and 1P_e_−1P_hh_ transitions. The electronic states of QDs are also shown in the right. **b** The PL spectra as a function of pump fluence. The QDs was spin-coated onto a glass substrate and pumped by 355 nm, 1.7 ns laser at room temperature. The PL spectra exhibit 1S emission at 625 nm, 1P emission at 595 nm and 1S ASE at 639 nm. **c** The 1S PL intensities (green line) and linewidths (yellow line) as a function of pump fluence. At ASE thresholds of 2.6 µJ cm^−2^, the emission intensity rises rapidly and super-lineally, accompanied by a significant narrowing of FWHM. **d** The dependence of the average QD occupancy <*N*> on pump intensity calculated considering multiexciton states of the order up to 6. **e** A schematic depiction of the bottom-emitting and top-emitting QLED structures and driving pulse signals. **f** Device temperature as a function of current density. **g** *J*-*V*-*L* curve of the bottom-emitting device. With effective thermal management, the device exhibits an injection current up to 1300 A cm^−2^. **h** The EL spectra as a function of current density. The EL spectrum at 802 A cm^−2^ is deconvolved into two Lorentzian bands that correspond to the 1S and 1P transitions

Figure 1a shows the spectroscopic properties of the CdSe-based QD gain medium. The photoluminescence (PL) peak is centered at 625 nm, corresponding to the band-edge 1S transition. A more detailed examination of the second derivative of the absorption spectrum reveals that the band-edge peak is a doublet, split by ~30 meV. We attribute the energy doublet to the 1S heavy hole (1S_hh_) and light hole (1S_lh_) states^6,11^, respectively. The second derivative of the absorption spectrum also exhibits a prominent peak at 2.12 eV, which can be attributed to the transition involving the 1P electrons and heavy hole states (1P_e_−1P_hh_ transition)^7,8^. The structure of the low-energy electronic states derived from above analysis is presented in the right side of Fig. 1a. At a low pump energy of 2.3 μJ cm^−2^, the 70-nm-thick QD film exhibits two emission peaks at 625 and 595 nm, corresponding to the 1S and 1P emission, respectively (Fig. 1b). As the pump energy increases, the PL spectra show another strong and sharp peak at 639 nm, which is regarded as the ASE at the 1S transition, since it rises rapidly and super-lineally (Fig. 1c), accompanied by a significant narrowing (from 25 to 3 nm) of the full width at half maximum (FWHM). The QD film exhibits a low 1S ASE threshold of 2.6 μJ cm^−2^, demonstrating its high potential for use in lasers. By performing the variable stripe length (VSL) ASE measurements (Supplementary Fig. S1), the 1S optical gain coefficient of a 70-nm-thick QD film is determined to be 690 cm^−1^, which is also regarded as the material gain coefficient (*G*_mat_) of the QDs due to a near-unity mode confinement factor of the measured film^10^. To directly compare surface and edge emission, we repeated the VSL measurements with PL collection from both the vertical and lateral directions. Due to the particle nature of the QDs, light propagating laterally is scattered by the QDs, resulting in the observed ASE signal in the vertical direction. As shown in Supplementary Fig. S2, by increasing the stripe length laterally, the gain length is increased, leading to enhanced PL intensity in the lateral direction. Meanwhile, due the presence of more QDs, the scattering probability increases with increasing stripe length. The combination of enhanced lateral PL and increased scattering events leads to stronger PL in the vertical direction as the stripe length increases. Due to scattering, photons emitted laterally or vertically undergo a similar number of scatterings and experience a similar path length over the gain region. This leads to similar PL spectra and gain coefficients being collected in the lateral and vertical directions, as illustrated in Supplementary Fig. S2.

### Electro-thermal co-design of high current injection QLEDs for population inversion

To evaluate how much current should be injected to induce the ASE, the average number of excitons per dot <*N*> at the ASE threshold was calculated. The QD films show a biexciton lifetime of 350 ps and a biexciton emission quantum yield of 13.8% (Supplementary Fig. S3 and Supplementary Table 1). Using these results, the lifetimes of multiexcitons (3~6) can be calculated^7,23^ (Supplementary Note 1), as presented in Supplementary Table 1. By inputting the multiexciton lifetimes, the number of excitons generated in each dot as a function of pump density can be calculated (Supplementary Note 2)^7,8^. As depicted in Fig. 1d, at the ASE threshold of 2.6 μJ cm^−2^, an average of 2.8 excitons are produced per QD. The corresponding current to generate <*N*> = 2.8 is 10.8 A cm^−2^, which sets the minimum threshold to achieve electrically pumped ASE.

The ASE threshold current is orders of magnitude higher than the typical operating current of QLEDs. At such high current levels, the device can generate large amounts of Joule heat, consequently limiting current injection, inducing nonradiative recombination or even causing catastrophic device failure. Therefore, to realize high current injection and stable operation of QLEDs at high current levels, effective thermal management is essential. To this end, QLEDs with a small emitting area of 0.01 mm^2^, fabricated on a Si substrate and driven by a 63 ns electrical pulse were developed (Fig. 1e). Spatially, by reducing the emission area, the Joule heat is greatly reduced and quickly dissipated, resulting in a significant increase in current (over 7.7 fold) and suppression of efficiency roll-off (Supplementary Fig. S6). Structurally, by using the Si with high thermal conductivity as a heat-sink substrate, the Joule heat can be effectively dissipated. Temporally, by driving the QLEDs with a 63 ns pulsed current, the accumulated heat can be greatly reduced. To evaluate the effect of thermal management on heat dissipation, the device temperature as a function of injection current was examined by analyzing the redshift of the electroluminescence (EL) spectra (Supplementary Fig. S7 and Supplementary Note 3)^8,20^. As shown in Fig. 1f, for regular devices (4 mm^2^ built on glass), the device temperature rises rapidly, reaching 470 K at a current of 3 A cm^−2^, leading to device catastrophic failure. In contrast, by reducing the device area to 0.01 mm² and using a Si heat sink substrate, the device temperature (305 K) is only slightly higher than the room temperature even at a high current of 7 A cm^−2^. Furthermore, by driving the devices with a 63 ns pulsed current, the device temperature is virtually the same as the room temperature at a high current up to 100 A cm^−2^. The details of the ns-pulsed driving and measurements are shown in Supplementary Fig. S8. The effective heat dissipation greatly suppresses the nonradiative recombination, the phonon scattering and carrier trapping/de-trapping events that frequently occur at high temperatures, leading to a significant increase in both efficiency and current (Supplementary Fig. S6). As a result, bottom-emitting (BE) QLED (Fig. 1e left side) with an injection current up to 1300 A cm^−2^ and a maximum brightness of 1.2 × 10^7 ^cd m^−2^ is achieved, as shown in Fig. 1g and Supplementary Fig. S9a. The presence of strong 1P emission in EL spectra (Fig. 1h) indicates that the injected current is high enough to induce a population inversion in QDs. The 1S and 1P emission intensity as a function of current is plotted in Supplementary Fig. S9b. Knowing the 1P/1S emission ratio, the number of excitons generated per QD can be extracted (Supplementary Note 4 and Supplementary Fig. S9b), which agrees well with the calculation results (Supplementary Fig. S9c). At an injection current of 600~1000 A cm^−2^, although a strong population inversion with 5~6 excitons per dot is achieved, no ASE is observed, suggesting that the optical gain is much lower than the optical losses, leading to a negative net gain and failure of the ASE.

### Optical design of cavity QLEDs for high modal gain

The optical losses intrinsically caused by the high absorption of the electrodes and the charge-conducting layers of the devices are unavoidable. Therefore, to achieve a net gain for ASE, the optical modal gain should be enhanced. To this end, a cavity structure (Fig. 1e, right) with the following features was delicately designed.

1. Top-emitting (TE) architecture which, on the one hand, allows the devices to be integrated with a Si heat sink and, on the other hand, eliminates the leakage of the optical field onto the substrate.
2. Planar FP cavity with metallic Ag mirrors that effectively resonates the QD emission with ease of fabrication. Compared to the BE device, the TE device exhibits a very sharp device reflection valley at 630 nm (Fig. 2a), implying that the cavity can strongly resonate the QD emission, consequently increasing the 630 nm photon density and facilitating the occurrence of ASE.

**Fig. 2:**
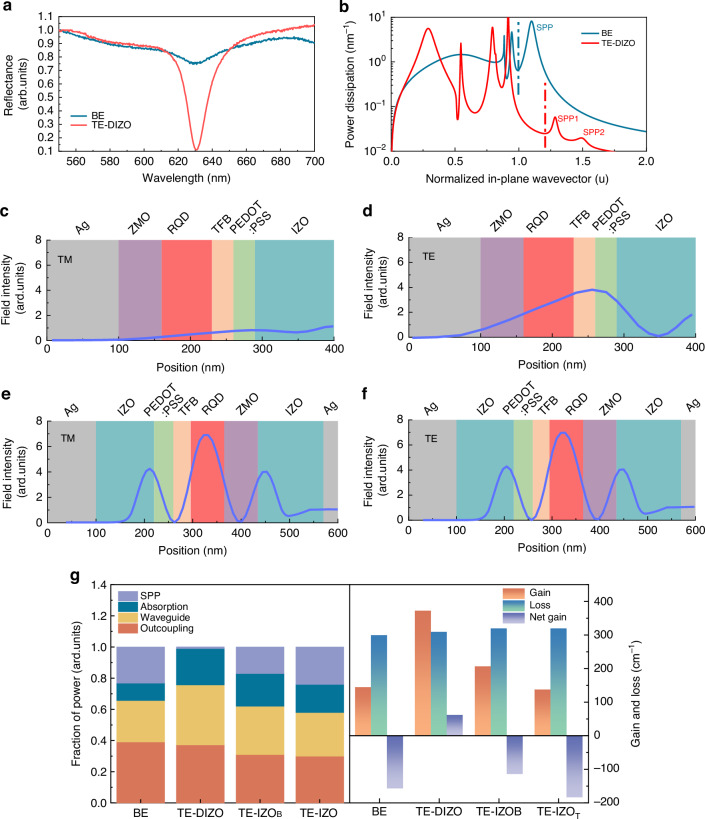
**Optical analysis of QLEDs with different structures**. **a** The reflectance spectra of BE and TE-DIZO devices. Compared to the BE device, the TE-DIZO device exhibit a sharp valley at 630 nm, indicating a strongly localized light field at 630 nm. **b** Power dissipation spectra of BE and TE-DIZO devices. The TE-DIZO device exhibits a significantly reduced SPP peaks and higher waveguide mode intensity. **c** Finite-difference time-domain (FDTD) simulations of TM_0_ and **d** TE_0_ mode propagation along the BE device. **e** FDTD simulations of TM_0_ and **f** TE_0_ mode propagation along the TE-DIZO device. Compared to the BE device, the TE-DIZO device exhibits a stronger optical field which is mainly localized in the QD layer. **g** Summary of fraction of power dissipation mode, optical modal gain, optical loss coefficient and optical net gain of TE_0_ mode at 630 nm of devices (Supplementary Note 6). Thanks to the key design of dual IZO layers, the TE-DIZO device exhibits the highest waveguide mode intensity and the highest optical modal gain, which enables it to show a positive net gain
3. Dual IZO phase tuning layers for improving the optical gain and reducing the optical losses. By using 140 and 120 nm IZO layers adjacent to the bottom Ag reflective electrode and the top semi-transparent Ag electrode, respectively, the SPP losses induced by the Ag electrodes are almost eliminated, as revealed by the power dissipation spectra shown in Fig. 2b (Supplementary Note 5). The reduced SPP modes are converted into the waveguide modes, which contribute to the enhancement of the optical field in QDs, as will be discussed later. In addition, the introduction of dual IZO layers allows the fine tune of optical field distribution, cavity resonance wavelength and power dissipation modes (Supplementary Figs. S11–S15) without compromising electrical performance. Furthermore, the incorporation of the dual IZO layers increases the separation between the QD layer and the Ag electrodes, thereby mitigating the quenching of QD excitons by Ag and reducing the losses. As revealed by the time-resolved (TR) PL spectra (Supplementary Fig. S16 and Supplementary Table 2), the use of IZO layers effectively restore the exciton lifetime from 19.43 to 23.15 ns, which is close to the intrinsic lifetime (23.35 ns) of QDs (Supplementary Table 2), implying a complete elimination of quenching. Overall, the introduction of dual IZO layers, with optimal thicknesses of 140 and 120 nm, respectively, perfectly enables the elimination of SPP losses, the resonance of QD emission, the enhancement of light outcoupling efficiency and the reduction of exciton quenching, which are essential for achieving a net gain.
4. A thicker QD layer of 70 nm, which ensures a sufficiently long light amplification path without deteriorating charge injection.

With these special designs, the optical modal gain of the resultant device (denoted as TE-DIZO) is greatly enhanced. Figure 2c–f shows the optical field distribution of the BE and TE-DIZO devices. The BE device shows a very weak transverse magnetic (TM) mode intensity (Fig. 2c), because the BE device has a strong SPP mode (Fig. 2b), which efficiently quenches the TM emission. In addition, both the TM and transverse electric (TE) modes are not concentrated within the QD layer (Fig. 2c, d), leading to a weak TE mode confinement factor (*Γ*_QD_) of 0.21 and a small modal gain (*G*_mod_ = *Γ*_QD_*G*_mat_) of 145 cm^−1^. In contrast, with the dual IZO design, the TE-DIZO device shows a significantly reduced SPP mode (Fig. 2b), and thus the TM mode is well preserved, which is essentially the same as the TE mode (Fig. 2e, f). Moreover, the design of a TE cavity and dual IZO layers converts the substrate and the SPP modes to the device waveguide mode (Fig. 2b), making the TE-DIZO device exhibit the maximum device waveguide mode (Fig. 2g). By fine tuning the distribution of device waveguide mode, the optical field can be maximally confined within the QD layer (Fig. 2e, f), making the TE-DIZO device exhibits a higher *Γ*_QD_ of 0.54 and a *G*_mod_ of 373 cm^−1^, which are more than double those of the BE device. The sharper device reflection valley at 630 nm (Fig. 2a) further confirms a more pronounced optical field localization at 630 nm for the TE-DIZO device.

Figure 2g summarizes the power dissipation mode, optical modal gain, optical loss coefficient and optical net gain at 630 nm of the BE, TE-DIZO and TE devices having only the bottom IZO (denoted as TE-IZO_B_) or the top IZO layer (denoted as TE-IZO_T_). Due to the strong field confinement within the QDs, the optical loss coefficients (*α*_loss_) of the TE-DIZO device (320 cm^−1^) is only slightly higher than that of the BE device and lower than those of the TE-IZO_B_ and TE-IZO_T_ devices, despite the introduction of an additional Ag or IZO layer. Among these four devices, the TE-DIZO device exhibits the lowest SPP mode intensity, the highest waveguide mode intensity and the highest modal gain, which is ascribed to the key design of the dual IZO layers. The TE-DIZO QLED structure strongly confines the optical field within the QD gain media, enabling the achievement of a positive net gain (*G*_*mod*_-*α*_*loss*_) and facilitating the realization of ASE in the lateral direction. The scattering of the lateral ASE could contribute to the ASE in the vertical direction, as will be discussed later.

### Realization of electrically pumped and surface-emitting ASE

**Fig. 3:**
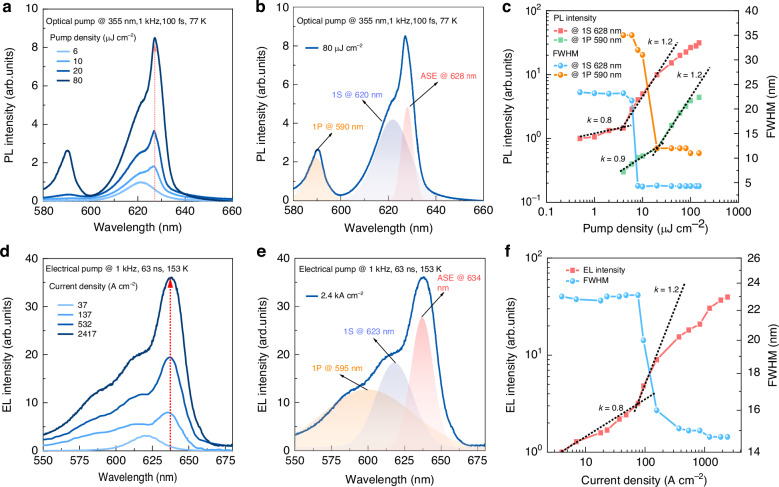
**Demonstration of optically and electrically pumped surface-emitting ASE in TE-DIZO devices**. **a**, **b** The PL spectra of TE-DIZO device pumped by a 355 nm fs laser at 77 K. The PL spectra can be deconvolved into three Lorentzian bands, corresponding to the 1P emission at 590 nm, 1S emission at 620 nm and 1S ASE at 628 nm. The 1S ASE at 628 nm increases rapidly and super-linearly, accompanied by a significant line narrowing. **c** The 1S (left, red squares) and 1P (left, green squares) PL intensities and linewidths (right, orange, and blue circle) as a function of pump fluence. These data exhibit typical signatures of the transition to ASE, signaled by the linewidth narrowing and the change in the log-log slope of the growth of the PL intensity. **d**, **e** The EL spectra of TE-DIZO device pumped by a 63 ns pulsed current at 153 K. The EL spectra can be deconvolved into three Lorentzian bands, corresponding to the 1P emission at 595 nm, 1S emission at 623 nm and 1S ASE at 634 nm. **f** The 1S (left, red squares) EL intensities and linewidths (blue circle) as a function of injection current. At the threshold current of 96 A cm^−2^, the EL at 634 nm increases rapidly and super-linearly (slope k changing from 0.8 to 1.2), accompanied by a narrowing of the linewidth, confirming the emergence of 1S ASE

The positive net gain implies a high probability of achieving ASE in TE-DIZO devices. To investigate such a probability, the TE-DIZO devices were optically pumped by a femtosecond laser at room temperature (RT). At a pump energy of 106 μJ cm^−2^, besides the 1S 630 nm and 1P 596 nm emission, a sharp emission peak at 640 nm appears (Supplementary Figs. S17a, S17b), which was also observed when the devices were electrically pumped at 2000 A cm^−2^ (Supplementary Figs. S17c, S17d). Such a sharp emission suggests the possible occurrence of ASE in the devices. Further increasing the pump energy leads to the burn out of the devices caused by the high instantaneous heat. To ensure stable operation of the devices at high pump energy, a cooling system was used, which mitigates the heat accumulation and, more importantly, greatly reduces the optical losses. On the one hand, the losses due to thermal-activate quenching are effectively suppressed, resulting in an enhanced and blue-shifted QD emission, as demonstrated in Supplementary Fig. S18a. On the other hand, the losses due to free electron absorption are greatly reduced (Supplementary Fig. S18b). As a result, at a low temperature of 77 K, the TE-DIZO device exhibits an ASE peak at 628 nm at relatively low pump energy, which increases rapidly and super-linearly with increasing pump energy, eventually overtaking the broad 1S peak, as shown in Fig. 3a (more data are shown in Supplementary Figs. S19a, S19b). The spectra at low temperature are blue-shifted and can be deconvolved into three Lorentzian bands that correspond to the 1P emission at 590 nm, 1S emission at 620 nm and ASE at 628 nm, as shown in Fig. 3b. Above the threshold energy of 8 and 60 μJ cm^−2^, respectively, the evolution of both 1S and 1P ASE is evident, characterized by both a faster (slope k increasing from 0.8 to 1.2) growth in intensity and a pronounced line narrowing (from 24 to 5 nm for 1S ASE), as revealed in Fig. 3a, c and Supplementary Figs. S19a and S19b. Impressively, similar ASE results are achieved by electrically pumping the TE-DIZO devices at 153 K. The sophisticated electrical and thermal design enables a high current of up to 2.4 kA cm^−2^ to be injected into the devices. As shown in Fig. 3d, e (more data are shown in Supplementary Fig. S19c), at high injection currents, the EL spectra exhibit three emission peaks at 595, 623 and 634 nm, corresponding to the 1P, 1S emission and 1S ASE, respectively, which are consistent with the results observed in optically pumped devices (Fig. 3a, b) or QDs (Fig. 1b). As shown in Fig. 3f and Supplementary Fig. S19d, when the current is increased from 96 to 2417 A cm^−2^, the 634 nm emission exhibits a super-linear (slope k increasing from 0.8 to 1.2) growth accompanied by a narrowing of the FWHM (from 24 to 15 nm), confirming the emergence of 1S ASE. Further increasing the current reduces the intensity growth slope and does not lead to a smaller FWHM or 1P ASE; this is because at high injection condition, a large amount of the injected electrons overflow from the QDs (Supplementary Fig. S20), resulting in the formation of leakage current that greatly reduces the emission efficiency of QDs^24^. Electron leakage at high current densities is experimentally quantified to account for ~17% loss in exciton occupancy (via multiexciton analysis at 1000 A cm^−2^). These results highlight leakage as a critical bottleneck for achieving higher ASE gain. By effectively confining the injected electrons within the QDs, a stronger and narrower 1S and 1P ASE could be achieved. We highlight that the optically or electrically pumped surface-emitting ASE can only occur in the specially designed TE-DIZO devices, while the BE or other TE devices pumped under the same conditions do not exhibit any sign of ASE (Supplementary Fig. S21).

Finally, it is worth noting that it is difficult to achieve surface-emitting ASE directly due to the thin thickness of the QD gain media, which results in a round-trip gain smaller than the ASE threshold. The observed surface-emitting ASE is resulted from the scattering of lateral ASE, which has also been observed in perovskite devices^5^. The TE-DIZO QLED structure developed here effectively confines the optical field within the QD gain media, thus facilitating ASE in the lateral direction. Due to the particle nature of the QDs, lateral ASE is effectively scattered, resulting in the observed ASE in the vertical direction. We are currently studying this scattering mechanism in detail, as well as its exact contribution to surface-emitting ASE.

**Fig. 4:**
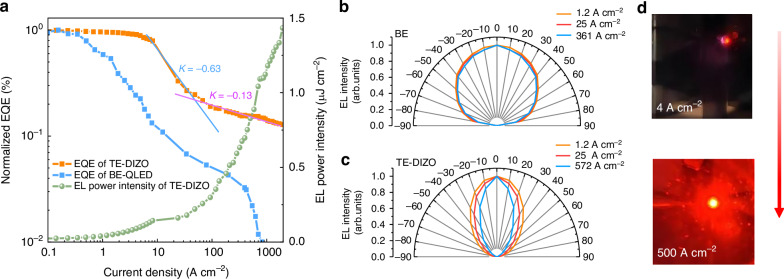
**Characterization of the output of the TE-DIZO device**. **a** Normalized EQEs of the BE (blue line) and TE-DIZO (orange line) devices and EL power (green line) output of the TE-DIZO device at 153 K. The TE-DIZO device exhibits suppressed efficiency roll-off due to the enhanced radiative recombination. In the high current ASE regime, the EQE roll-off slope is smaller than that before the onset of ASE. The maximal output power density reaches 1.3 μJ cm^−2^. **b**, **c** The angularly dependent EL emission of BE and TE-DIZO devices. The TE-DIZO device shows a strong emission directionality with a smaller beam divergence. **d** Photographs of the TE-DIZO device operating at different current density

Figure 4a shows the normalized external quantum efficiency (EQE) of the TE-DIZO and BE devices, highlighting that the TE-DIZO device demonstrates a notably weaker efficiency roll-off at high currents. The suppressed efficiency roll-off is mainly attributed to the accelerated radiative recombination in TE-DIZO device, which enables the reduction of excitons quenching by nonradiative processes such as Auger^25^ and thermal-induced quenching^20^. Moreover, in the high current ASE regime, the EQE roll-off slope (k = 0.13) is smaller than that (k = 0.63) before the onset of ASE, indicating the light amplification process in the ASE regime. We also compared the normalized EQE at room temperature and at 153 K (Supplementary Fig. S22), and the EQE roll-off at 153 K is significantly smaller. The suppressed efficiency roll-off leads to a high photon energy output of 1.4 μJ cm^−2^. Figure 4b, c evaluates the emission directionality of the devices. Compared to the stable Lambertian emission of the BE device, the TE-DIZO device shows a strong forward emission directionality. Moreover, by increasing the pump current, the emission directionality is further enhanced, reaching a 1.75-fold improvement with a small emission angle of ±20 degrees at a current of 572 A cm^−2^. Despite the small emitting area of only 0.01 mm^2^, the TE-DIZO device exhibits very bright and directional emission after the onset of ASE, as shown in Fig. 4d.

## Conclusion

In conclusion, electrically pumped and surface-emitting ASE from solution-casted colloidal QDs has been demonstrated. This achievement has been enabled by the electro-thermal-optical co-design of a QLED that simultaneously exhibits high current injection and positive net optical gain. The electro-thermal co-design enables the Joule heat to be effectively dissipated and, allows a high current up to 2000 A cm^−2^ to be injected into the QLED, resulting in a strong population inversion with maximum 5~6 excitons per dot and leading to the strong emission from both 1S and 1P transitions. The delicately optical design of a top-emitting FP cavity effectively resonates the QD emission. The key design of dual IZO layers adjacent to the metallic electrodes significantly reduces the SPP losses and greatly enhances the waveguide mode, enabling a higher mode confinement factor of 0.56 and a 2-fold enhancement of optical modal gain, eventually resulting in a positive net gain and the realization of 1S and 1P optically pumped ASE. The above electro-thermal-optical co-design allows the resultant TE-DIZO device to demonstrate electrically pumped and surface-emitting ASE with strong directionality, high intensity and narrow linewidth at a threshold current of 94 A cm^−2^. By further reducing electron leakage at high currents, stronger electrically pumped ASE or even lasing could be achieved, which could eventually leads to the realization of VCSEL diodes.

## Methods

### Materials

Colloidal CdSe-QDs were purchased from Suzhou Xing-shuo Nanotech Co., Ltd. ZnMgO nanoparticles in solution were purchased from Guangdong Poly OptoElectronics Co., Ltd. TFB HTL (TFB=poly (9,9-dioctylfluorenyl-2,7-diyl)-alt-(4,4′-(N-(4-butylphenyl))) were purchased from American Dye Source Co., Ltd.. PEDOT:PSS (CLEVIOS P AI4083) was purchased from Xian Polymer Light Technology Corp. The chemicals chlorobenzene and octane were obtained from Aladdin Industrial Corp., while absolute ethanol was obtained from Shanghai Ling Feng Chemical Reagent Co., Ltd. The ITO (ITO = indium tin oxide, PEDOT:PSS = poly-(ethylenedioxythiophene)/polystyrenesulfonate) glass with a sheet resistance of 20 Ω sq^−1^ was obtained from Wuhu Jinghui Electronic Technology Co., Ltd.

### Device structures

TE-DIZO: Si/Ag (100 nm) /IZO (140 nm)/PEDOT:PSS (45 nm)/TFB (35 nm)/QD (70 nm)/ZnMgO (65 nm)/IZO (120 nm)/Ag (20 nm)

TE-IZO_B_: Si/Ag (100 nm)/IZO (120 nm)/PEDOT:PSS (45 nm)/TFB (35 nm)/QD (70 nm)/ZnMgO (65 nm)/Ag (20 nm)

TE-IZO_T_: Si/Ag (100 nm)/PEDOT:PSS (45 nm)/TFB (35 nm)/QD (70 nm)/ZnMgO (65 nm)/IZO (130 nm)/Ag (20 nm)

BE: glass/IZO (110 nm)/PEDOT:PSS (45 nm)/TFB (35 nm)/QD (70 nm)/ZnMgO (65 nm)/Ag (100 nm)

Abbreviations: IZO = In_2_O_3_:ZnO = 90:10 wt%, PEDOT:PSS = poly-(ethylenedioxythiophene)/polystyrenesulfonate, TFB = poly (9,9-dioctylfluorenyl-2,7-diyl)-alt-(4,4′-(N-(4-butylphenyl)

### Device fabrication

For TE-DIZO devices, the Si substrates were rinsed in ultrasonic detergent and deionized water for 30 min in sequence and then dried in an oven at 60 °C for 30 min. Then the cleaned substrates were transferred to the magneton sputtering system and the bottom reflective cathode made of Ag (100 nm)/IZO (140 nm) was deposited at a pressure of 0.45 Pa with a power of 50 W and an Ar flow of 20 sccm. After that, the cathode was treated with O_2_ plasma for 5 min, followed by spin-coating PEDOT:PSS layer at 3000 rpm for 45 s and baked at 130 °C for 20 min in air. The TFB solution (8 mg mL^−1^ in chlorobenzene) was then spun-cast on the top of PEDOT:PSS layer at 3000 rpm for 45 s and baked at 120 °C for 15 min in nitrogen-filled glove box. Afterwards, 20 µL of QD solution (25 mg ml^−1^ in octane) was spun-cast at 3000 rpm for 30 s and baked at 100 °C for 15 min. This procedure was repeated twice to produce a 70 nm QD film. Afterward, ZnMgO NPs (30 mg mL^−1^ in ethanol) were spun-cast on QDs at 3000 rpm and annealed at 100 °C for 10 min. Then, the  120 nm transparent IZO anode was deposited by magneton sputtering system at a pressure of 0.45 Pa, a power of 50 W and an Ar flow of 20 sccm. Finally, a thermal evaporation Ag (20 nm) deposited at 4 × 10^−1^ Pa and 5 Å s^−1^ was used as the reflective anode. For TE-IZO_B_ and TE-IZO_T_ devices, expect for the elimination of one IZO layer, all layers are the same with those of TE-DIZO. For BE devices, expect for the cathode and anode, all layers are the same with those of TE-DIZO. The IZO ( 110 nm) deposited as above description was used as the bottom transparent anode, and a thermal evaporation Ag (100 nm) deposited at 4 × 10^−4 ^Pa and 5 Å s^−1^ was used as the reflective cathode.

### Device characterization

The thicknesses of all films were measured by a Bruker DektakXT Stylus Profiler. The evaporation rates and the thicknesses of Ag electrode, were in situ monitored by a quartz crystal microbalance. The TRPL spectra was measured with an Edinburgh instruments FS5 Fluorescence Spectrometer. EL spectra of QLEDs were measured by a fiber-optic spectrometer (USB 2000, Ocean Optics) in the normal direction, and the *J-V-L* characteristics of QLEDs were assessed by using a dual-channel Keithley 2614B programmable source meter with a PIN-25D calibrated silicon photodiode (PD) under ambient conditions. Thermal images were captured with an Optotherm IS640 infrared camera. To carry out the temperature-dependent experiments, a custom designed temperature-controllable probe station was used. Liquid nitrogen is used to control the temperature with a minimum accuracy of 0.1 °C. Temperature-dependent EL spectra were obtained using this probe station. The ns-pulsed module was custom-developed to support the high voltage adjustable nanosecond pulse electrical signal output from 10 to 150 ns within a voltage range from 16 to 36 V. Repetition frequency was set at 1 kHz when measuring the pulsed EL spectra. The output ports are soldered directly to the device electrodes to reduce transmission resistance, and the circuit board is equipped with a 50 mOhm current detection module for transient current monitoring. A Si-APD (THORLABS, APD120A2/M) and an oscilloscope (Tektronix, TBS1102) were used to detect the pulsed optical signals generated by the devices.

### Optical measurements

Optical absorption measurements: Optical absorption spectra were measured using an integrating sphere module and a UV/Vis spectrometer. For calibration, a bare glass was measured and the acquired spectrum was used as a background signal.

Optically excited ASE: A device or 70 nm QD film was mounted into a cryostat and characterized at either room or liquid helium temperature. Optical excitation was conducted via an opening in the sample holder through the transparent electrode. The 100-fs, 355-nm pump pulses (1-kHz repetition rate) were used as excitation source. A CaF_2_ lens was used to focus the beam onto the devices. The excited photoluminescence (PL) of the active area was collected from the surface of the sample and spectrally resolved using a Czerny-Turner spectrograph (Acton SpectraPro 300i) coupled to a CCD camera (Roper Scientific).

VSL measurements: VSL measurements were conducted using the same configuration as in the ASE experiments with the excited stripe-shaped area of a varied length. The stripe length (*x*) was varied in ~0.5 mm steps using a razor blade mounted onto a translation stage. Optical gain (*G*) was obtained by fitting the measured edge-emitted PL intensity (*I*) to *I* *=* *A[exp(Gx)* *–* *1]/G* *+* *Bx*, where *A* and *B* were *x*-independent constants.

Biexcition lifetime measurements: A QD sample, prepared as a spun-coated film, was excited by 355-nm, 100-fs pulses focused into a spot. The emitted PL was collected in the direction normal to the sample plane, spectrally dispersed with a Czerny-Turner spectrograph and detected with an avalanche photodiode coupled to a time-correlated single-photon counting system.

### Optical simulations

To determine the TE_0_ and TM_0_ mode characteristics of QLED device configurations, we used Finite-difference time-domain (FDTD) solver and Finite Difference Eigenmode (FDE) solver in Ansys Lumerical. The studied LED structures were considered as an ideal planar slab waveguide with zero roughness. All computation was conducted in 2D domain.

## Supplementary information

Supplementary Information for Electrically pumped surface-emitting amplified spontaneous emission from colloidal quantum dots

## Data Availability

The data that support the findings of this study are available from the corresponding author upon reasonable request.
