# Supplementary material for: Electrically pumped surface-emitting amplified spontaneous emission from colloidal quantum dots
Source: Light Sci Appl. 2025 Aug 19;14:279. doi: 10.1038/s41377-025-01972-1 (PMC12365173; doi:10.1038/s41377-025-01972-1)
Supplement: Supplementary file 1 — Supplementary Information for Electrically pumped surface-emitting amplified spontaneous emission from colloidal quantum dots [file 41377_2025_1972_MOESM1_ESM.docx]

# Supplementary Information for

**Electrically pumped surface-emitting amplified spontaneous emission from colloidal quantum dots**

Fengshou Tian, Tianhong Zhou, Xuanyu Zhang, Rui Chen, Shuming Chen*

State Key Laboratory of Quantum Functional Materials, Department of Electrical and Electronic Engineering, Southern University of Science and Technology, Shenzhen 518055, P. R. China

* Corresponding author: Shuming Chen ([chen.sm@sustech.edu.cn](mailto:chen.sm@sustech.edu.cn))

**Supplementary Note 1**

**Calculation of radiative rates**

We have utilized the calculated occupation factors to ascertain the rates of radiative transitions for specific electron-hole state pairs ^[1]^. We employed the most general condition under which the onset of population inversion occurs for a specific pair of electron and hole states:

$f_{e,i}+f_{h,j}=1 （1）$

where $f_{e,i}$ and $f_{h,j}$ are the occupation factors of the *i*-electron and *j*-hole states linked to temperature (*T*). The $f_{e,i}$ and $f_{h,j}$ are determined by the Fermi-Dirac distribution function:

$f_{e,i}=\frac{1}{1+e^{\frac{E_{e,i}-\mu_{e}}{kT}}} （2）$

$f_{h,j}=\frac{1}{1+e^{\frac{E_{h,j}-\mu_{h}}{kT}}} （3）$

where $E_{e,i}$ and $E_{h,j}$ are the electron and hole state energies, respectively, and $\mu_{e}$ and $\mu_{h}$ are the chemical potentials of electrons and holes, respectively.

For a QD is occupied with $N_{e}$ electrons and $N_{h}$ holes, the $N_{e}$ and $N_{h}$ can be expressed by:

$$N_{e}=\sum_{i} g_{e,i}f_{e,i} （4）$$

$$N_{h}=\sum_{j} g_{h,j}f_{h,j}（5）$$

where $g_{e,i}$ and $g_{h,j}$ are the degeneracy factors of the *i*-electron and *j*-hole states, respectively. For the S- and P- states, the degeneracy factors$g_{e,i}$ and $g_{h,j}$ are equal to 2 and 6, respectively. By applying the$E_{e,1s_{e}}=-4.0 eV$, the specific energy level of a QD can be calculated through Fig. 1a of main text. For the specific electronic configuration when a QD is occupied with $N_{e}$ electrons and $N_{h}$ holes, the $\mu_{e}$ and $\mu_{h}$ can be computed from equation (4) and (5).

The radiative rate of a transition coupling the *i*-electron to the *j*-hole state ($\gamma_{ij,r}$) is directly proportional to the product of the corresponding occupation factors:

$$\gamma_{ij,r}\propto f_{e,i}\times f_{h,j} （6）$$

By employing the occupation factors depicted in Supplementary Fig. 3a and the statistical scaling of multicarrier lifetimes as described in equations (8)-(10) ^[2]^, we can derive the radiative decay rates for neutral multiexcitons, ranging from biexcitons to six-excitons (denoted as 2X, 3X, ..., 6X):

$$\tau_{2r}{=1}/4\tau_{1} (7)$$

$$\tau_{NA}=4\tau_{2A}\left[ N^{2}\left( N-1 \right) \right]^{-1} (8)$$

$$\gamma_{NX}=1/{\tau_{NX}}=1/{\tau_{NX,r}}+1/{\tau_{NX,A}} (9)$$

where $\tau_{N, r}$ is radiative lifetime of N-excitons, $\tau_{N, A}$ is Auger lifetimes of N-excitons, $\gamma_{NX}$ is emission rates of *N*-excitons, and *N* is the number of excitons. The calculation results are summarized in Supplementary Table 1.

**Supplementary Note 2**

**Calculation of exciton occupation of QDs as a function of pump density**

The rate equations used in our calculations is (*k* = 0 to 6) ^[1, 4]^

$$\frac{dn_{k}}{dt}=-g_{E}n_{k}-\frac{n_{k}}{\tau_{k}}+g_{E}n_{k-1}+\frac{n_{k+1}}{\tau_{k+1}} （10）$$

we take into account exciton multiplicities from 0 to 6 with corresponding probabilities *n_0_* to *n_6_* (*n_0_* + … + *n_6_* = 1) and lifetimes $\tau_{1}$ to $\tau_{6}$. Here $g_{E}$ is the excitation rate given by $g_{E}={I_{PL}\sigma}/{h\nu_{pump},}$ where $I_{PL}$is the pump intensity, $\sigma$ is the absorption cross section (1ⅹ10^-12^ cm^2^), and $h\nu_{pump}$ is the excitation photon energy. In the steady state (${dn_{k}}/{dt}=0$), the solution of above equation is:

$$n_{0}=\frac{1}{1+g_{E}\tau_{1}+\ldots+g_{E}^{6}\tau_{1}\tau_{2}\tau_{3}\tau_{4}\tau_{5}\tau_{6}} （11）$$

$$n_{k}=g_{E}^{k}\tau_{1}\cdots\tau_{6} (18)$$

The average QD occupancy $<N_{PL}>$ can be expressed as:

$$<N_{PL}>= \frac{g_{E}\tau_{1}+2g_{E}^{2}\tau_{1}\tau_{2}+\ldots+6g_{E}^{6}\tau_{1}\tau_{2}\tau_{3}\tau_{4}\tau_{5}\tau_{6}}{1+g_{E}\tau_{1}+2g_{E}^{2}\tau_{1}\tau_{2}+\ldots+6g_{E}^{6}\tau_{1}\tau_{2}\tau_{3}\tau_{4}\tau_{5}\tau_{6}} （12）$$

Exciton occupation of QDs as a function of current density:

We apply the above model to the current density. The average QD occupancy can be expressed as:

$$<N_{EL}>= \frac{g_{E}\tau_{1}+2g_{E}^{2}\tau_{1}\tau_{2}+\ldots+6g_{E}^{6}\tau_{1}\tau_{2}\tau_{3}\tau_{4}\tau_{5}\tau_{6}}{1+g_{E}\tau_{1}+2g_{E}^{2}\tau_{1}\tau_{2}+\ldots+6g_{E}^{6}\tau_{1}\tau_{2}\tau_{3}\tau_{4}\tau_{5}\tau_{6}} （13）$$

$$g_{E}=J/\rho（14）$$

where *J* is a current density, *ρ* is an areal QD density and *τ_i_* is the lifetime of the *i_th_* multiexciton. The areal QD density *ρ* can be calculated by measuring the average diameter of QDs, which is 11.7 nm, as shown in Supplementary Figure 4. The lifetime of the *i_th_* multiexciton can be obtained from Supplementary Table 1.

**Supplementary Note 3**

**Calculation of device temperature**

As shown in Supplementary Fig. 5(a) and (e), with increasing current density, the emission spectra show the obvious red-shift and the broadening of the emission line-width. Commonly, the red-shift of the emission peak is caused by the temperature-induced bandgap shrinkage, and the broadening of the emission line-width is ascribed to the enhanced carrier-phonon scattering at elevated temperature. We use Varshni relation to estimate the temperature variation of the device under different current densities ^[5]^:

$$E_{g}\left( T \right)=E_{g0}-\frac{\alpha T^{2}}{\left( T+\beta\right)} （15）$$

where $E_{g0}=2.37 \mathrm{eV}$ is the energy gap at 0 K, $\alpha=4.7\times{10}^{-4} eV/K$ is the temperature coefficient, and $\beta=51.1 K$ is a parameter related to the Debye temperature of the material.

**Supplementary Note 4**

**Modelling of exciton number of QDs from spectra intensity of 1S and 1P.**

In order to evaluate the exciton number of QDs, we assume the emission intensity is proportional to the product of multi-exciton occupancy and transition probability ^[1, 4]^. The ratio of 1s to 1p emission intensity is defined as:

$$r=\frac{I_{1P}}{I_{1S}} （16）$$

where *I*_1_*_P_* and *I*_1_*_S_* represents the emission intensity from the 1P and 1S states, respectively.

Suppose the exciton number *N* follows a Poisson distribution *P*(*N*) throughout the entire QD ensemble.

$$P\left( N \right)=\frac{\left\langle N \right\rangle^{N}e^{-\left\langle N \right\rangle}}{N!} （17）$$

$\left\langle N \right\rangle$represents the average number of excitons.

The emission intensity $I_{1P}$and $I_{1S}$ can be determined by the distribution of multi-excitons and the number of excitons:

$$I_{1S}\propto P\left( N \right)\times N_{1} （18）$$

$$I_{1P}\propto P\left( N \right)\times N_{2} （19）$$

where $N_{1}$ and $N_{2}$ represents the exciton number of the 1P and 1S states, respectively.

Considering the transition probability of *N* excitons, the probability is proportional to the number of excitons of 1S and 1P:

$$P_{1S}\propto N-1 （20）$$

$$P_{1P}\propto N-2 （21）$$

The ratio of 1S to 1P emission intensity *r* is:

$$r=\frac{\int_{N=2}^{\infty} P\left( N \right)\times{(N-2)}^{2}dN}{\int_{N=1}^{\infty} P(N)\times{(N-1)}^{2} dN} （22）$$

*r* can be approximately obtained as:

$$r=\frac{{(N-2)}^{2}}{12} （23）$$

**Supplementary Note 5**

**Optical modeling of QLED devices**

We use the classical formalism describing dipole radiation to simulate the light emission of QLED devices ^[6-9]^. In this model, the emitter is treated as a forced, damped, electric dipole oscillator located in a planar multilayer structure, as shown in Supplementary Figure 9. The dipole is located in region 1 and sandwiched between the reflection region 2 and the transmission region 3. The wave generated by dipole radiation is reflected back and forth by region 2 and region 3. If the reflected wave is in phase, the emission increases, and the exciton decay rate increases. Otherwise, the emission is suppressed, and the exciton decay rate decreases.

The total power consists of three parts, for vertical dipoles coupling to TM waves, and for horizontal dipoles coupling to TM and TE waves, separately, which can be calculated respectively.

The unitless in-plane wavevector (denoted as *u*) in Figure 2b is defined as the normalized component of the wavevector parallel to the device layers:

$$u=\frac{k_{x}}{k_{0}}$$

where *k_x_* is the in-plane wavevector component, and *k_0_=2π/λ* is the free-space wavevector. This normalization allows *u* to represent the ratio of the in-plane momentum to the free-space momentum, which is critical for analyzing optical modes (e.g., waveguide, SPP) in layered structures. Values of *u*>1 correspond to evanescent waves (e.g., SPP modes), while *u*<1 represent propagating modes (e.g.,waveguide). This framework is standard for analyzing optical modes in planar microcavities and is consistent with prior studies ^[6-9]^.

For the vertical dipole, the power intensity of the generated TM wave at a wavelength of $\lambda$ and a normalized in-plane wavevector u is:

$$K_{TMv}=\frac{3}{2}Re\left[ \frac{u^{3}}{\sqrt{1-u^{2}}}\frac{\left( 1+a_{1,2}^{TM} \right)\left( 1+a_{1,3}^{TM} \right)}{1-a_{TM}} \right] （24）$$

For the horizontal dipole oriented in the plane, the power density of the generated TM and TE waves at a wavelength of $\lambda$ and a normalized in-plane wavevector u is:

$$K_{TMh}=\frac{3}{4}Re\left[ u\sqrt{1-u^{2}}\frac{\left( 1-a_{1,2}^{TM} \right)\left( 1-a_{1,3}^{TM} \right)}{1-a_{TM}} \right] （25）$$

$$K_{TEh}=\frac{3}{4}Re\left[ \frac{u}{\sqrt{1-u^{2}}}\frac{\left( 1+a_{1,2}^{TE} \right)\left( 1+a_{1,3}^{TE} \right)}{1-a_{TE}} \right] （26）$$

where *Re*[…] represents the real part of the complex.

Moreover, the factor $1\pm a_{12,13}$ in the equations (24)~(26) describes the wide-angle interference. The factor $1-a_{TM,TE}$ in the equations describes the multiple-beam interference, Furthermore,

$$a_{1,2}^{TM,TE}=r_{1,2}^{TM,TE}\exp\left( 2jk_{z,e}z_{1,2} \right) （27）$$

$$a_{1,3}^{TM,TE}=r_{1,3}^{TM,TE}\exp\left( 2jk_{z,e}z_{1,3} \right) （28）$$

$$a_{TM,TE}=a_{1,2}^{TM,TE}*a_{1,3}^{TM,TE}=r_{1,2}^{TM,TE}*r_{1,3}^{TM,TE}*\exp\left( 2jk_{z,e}d \right) （29）$$

where $r_{1,2}^{TM,TE}$ and $r_{1,3}^{TM,TE}$ are the reflection coefficients of the bottom interface (between region 1 and 2) and the top interface (between region 1 and 3), respectively. $z_{1,2}and z_{1,3}$are the distances of the emitting dipoles from the bottom and the top interfaces, respectively, and d is the thickness of the emitting layer.

The power dissipation spectrum $K_{u}$distinguishes three regions:

(1) Air-radiated modes ($0<u<1/{n_{e}}$): Propagating waves transmitted into air.

(2) Substrate-trapped modes​​ ($1/{n_{e}}<u<{n_{sub}}/{n_{e}}$): Waves guided in the substrate.

(3) Evanescent/SPP modes​​ ($u>1$): Non-propagating waves coupled to lossy SPPs.

The fractional power for each mode is calculated by integrating *K(u)* over the corresponding *u*-range.

The total radiated power *F(λ)* (Purcell factor) modifies the emitter’s radiative decay rate and quantum yield *η_rad,cav_*. The final light enhancement *η_enhancement_* combines cavity-modified QY and far-field coupling efficiency *η_out_*.

**Supplementary Note 6**

**Mode simulations**

To determine the fundamental transverse electric mode (TE_0_) and transverse magnetic mode (TM_0_) characteristics of device configurations, we used a Finite Difference Eigenmode (FDE) solver in Ansys Lumerical. The refractive indices, as depicted in Supporting Information Fig. 8a, were incorporated into the model. However, the absorption of the QD layer was disregarded and set to zero, mimicking a transparency condition, which enabled the loss assessment to be solely from the surrounding layers. The mode confinement factor (*Γ*) was calculated using the following equation:

$$\Gamma=\frac{\int_{QD} {|E|}^{2}dz}{\int{|E|}^{2}dz} （30）$$

where *|E|^2^* is the electric field intensity of the fundamental TE_0_ mode, z is the vertical coordinate. The optical propagation loss *α* (in cm^−1^) was calculated from the following equation:

$$\alpha=-\frac{4\pi}{\lambda}Im \left( n_{eff} \right) （31）$$

where *λ* is the wavelength, *n_eff_* is the effective refractive index and *Im* denotes the imaginary component.

**Supplementary Figures**


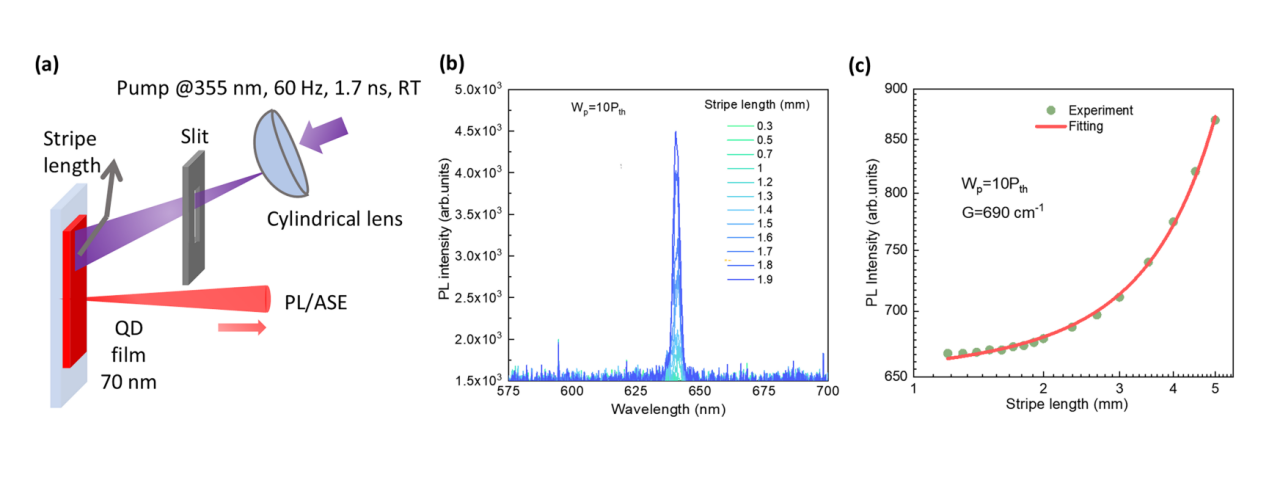


**Supplementary Figure 1. Variable stripe length (VSL) measurements.** (a) VSL measurements were conducted using the same configuration as in the ASE experiments with the excited stripe-shaped area of a varied length. The QD film (70 nm thick) was excited at 355 nm with 1.7 ns pulses at 60 Hz. The pump beam was focused with a cylindrical lens onto the sample into a narrow stripe which is perpendicular to the sample edge. The stripe length was varied from 0.02 to 5 mm using a razor blade translated in the direction perpendicular to the pump beam. (b) A series of PL spectra is collected from the surface of the films and the PL spectra exhibits ASE peak. (c) The dependence of the 1S ASE signals on stripe length. Optical gain (G) was obtained by fitting the measured PL intensity (*I*) to *I = A [exp (Gx) –1]/G* + *Bx*, where *A* and *B* were *x*-independent constants. By fitting, the *G, A, B* are determined to be 690 cm^-1^, 0.0391, 0.64, respectively.


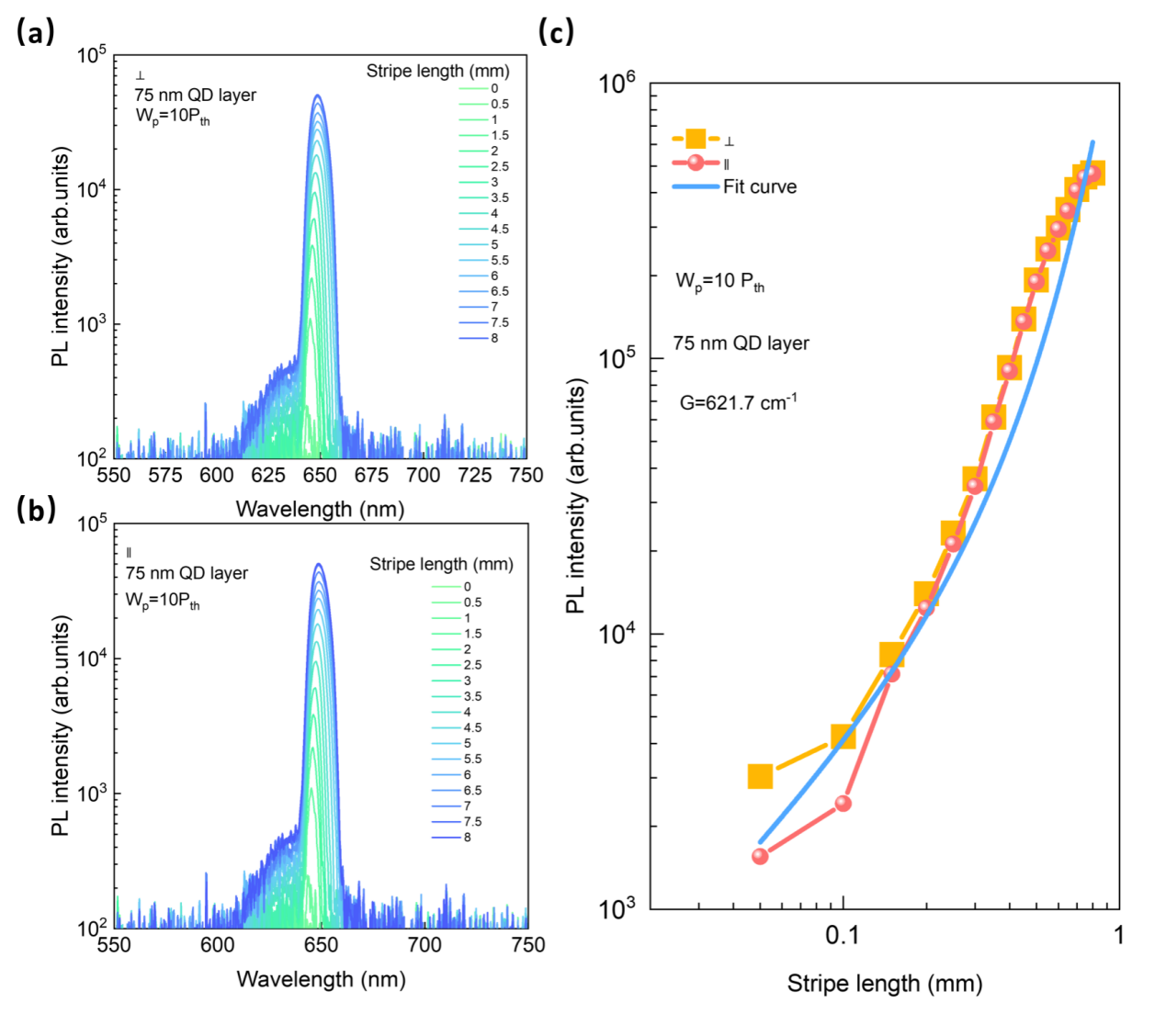


**Supplementary Figure 2. The comparison of the VSL measurements of a 75 nm QD layer with PL collection from edge and surface.** (a) The PL spectra collected from surface emission. (b) The PL spectra collected from edge emission. (c) The gain coefficients of the edge-emitting ASEs differ very little from those of the surface emission.


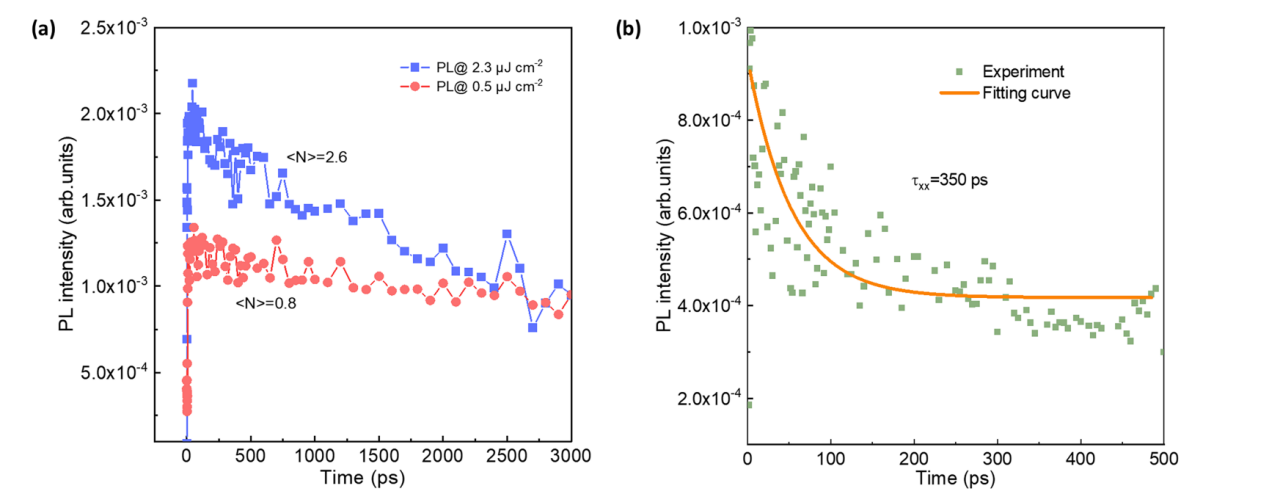


**Supplementary Figure 3. The total decay rate of a given neutral state.** (a) PL dynamics of the QD films. PL dynamics of the QD film sample measured at the excitation levels which correspond to the average per-dot excitonic occupancies *<N>*= 0.8 (red line) and 2.6 (blue line). The sample was excited using 100 fs, 355-nm pulses with the pulse repetition rate of 1 kHz. (b) The dynamics obtained by subtracting the two traces in panel (a) yield information on biexciton recombination. By fitting the PL decay curve using a single-exponential equation$y=y_{0}+A_{1}*exp[-\left( x-x_{0} \right)/t_{1}]$, the biexciton lifetime $t_{1}$ is determined to be $t_{1}$=350 ps (corresponding to $y_{0}$=2.25ⅹ10^-4^, $A_{1}$=4.64ⅹ10^-4^, $x_{0}$=3.3ⅹ10^-3^ ps).


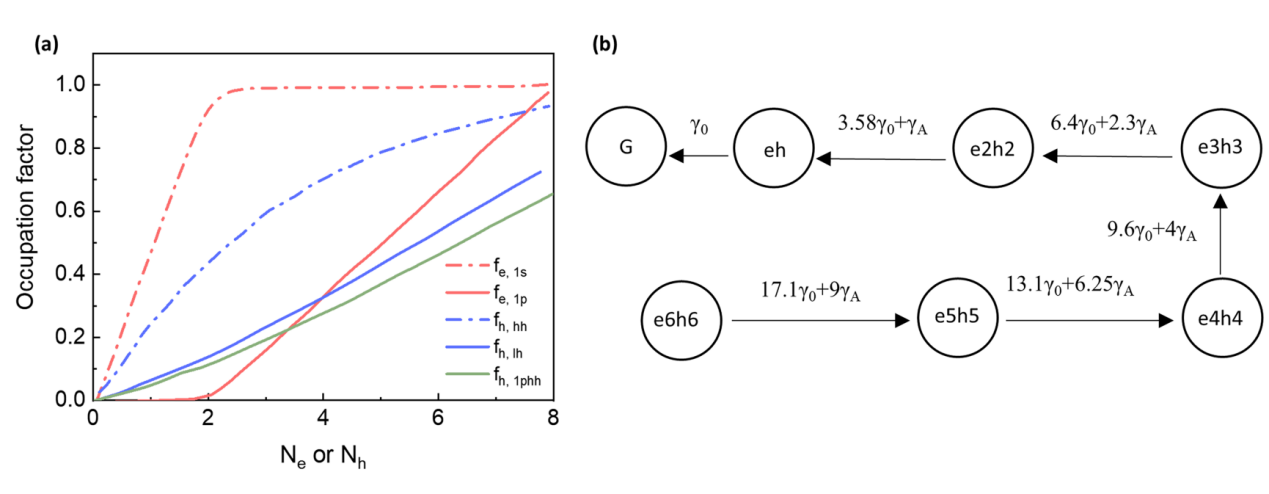


**Supplementary Figure 4.** (a) Calculated occupation factors of the various electron (*f_e,i_*, red) and hole (*f_h,j_*, blue & green) states as a function of the total number of electrons (*N_e_*) or holes (*N_h_*) based on Note 1. (b) Schematic illustration of the mode used in calculations of the multi-exciton lifetimes. *G* and *e_x_h_x_* represent the ground state of the a QD and a QD filled with *x* excitons, respectively. The total decay rate of a given neutral state is calculated as a sum of the radiative and nonradiative Auger decay rates. Radiative decay rates are computed based on the electron and hole occupation factors as detailed in Note 1 and the Auger decay rates are found from the measured biexciton lifetime.


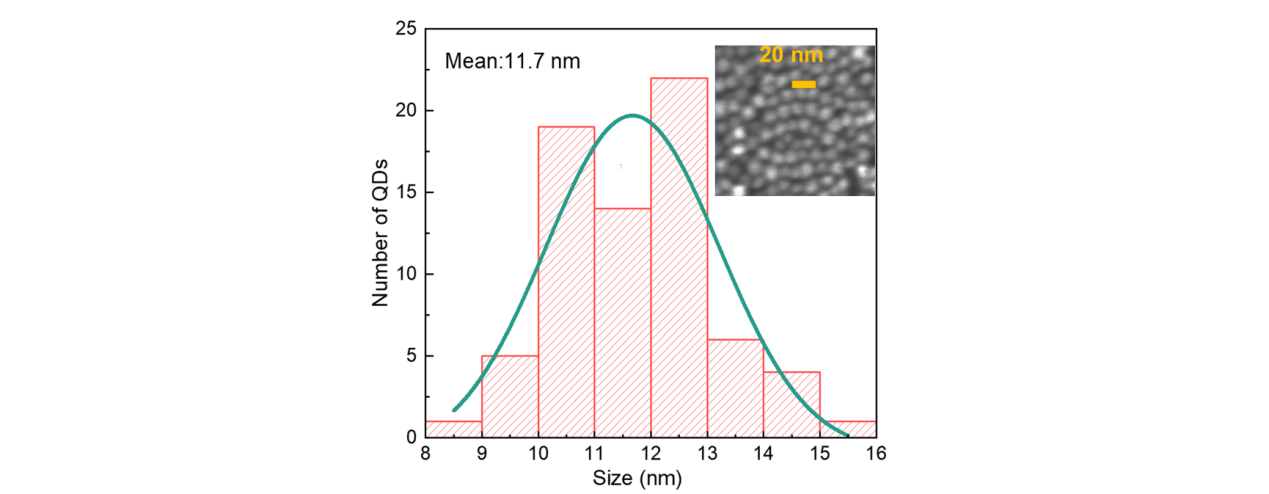


**Supplementary Figure 5.** The average diameter of QDs and SEM scan of the QD film (insert). The average diameter of QDs is 11.7 nm.


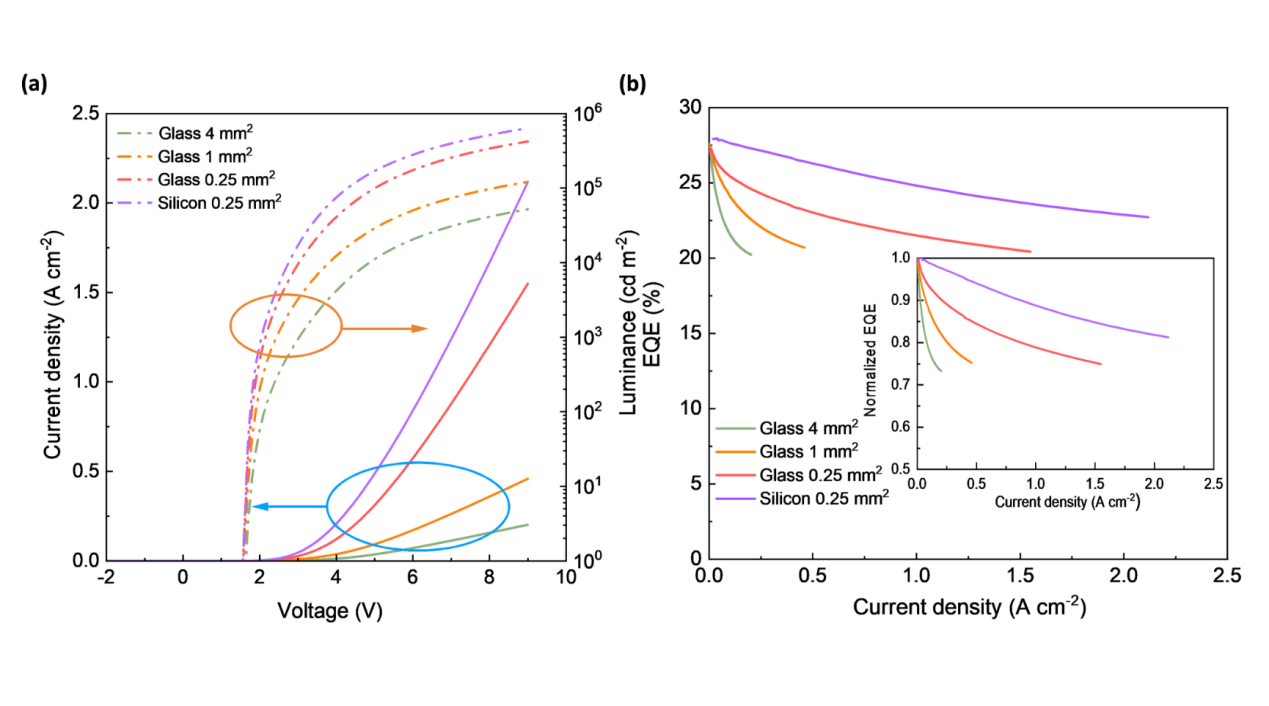


**Supplementary Figure 6. Comparison of electrical properties of devices with different areas.** Device structures for the green line, orange line and red line: Glass/ITO (70 nm)/PEDOT:PSS (45 nm)/TFB (30 nm)/RQD (30 nm)/ZMO (65 nm)/Al. Purple line: Silicon/Ag (100 nm)/IZO (5 nm)/PEDOT:PSS (45 nm)/TFB (30 nm)/RQD (30 nm)/ZMO (65 nm)/Al (2 nm)/IZO (50 nm). (a) Comparison of *J-V-L* curves of different devices. (b) Comparison of EQE of different devices. It is evident that reducing the device area results in higher current densities, and smaller areas significantly reduce EQE roll-off. The use of silicon substrate with enhanced heat dissipation further increases current densities and reduces EQE roll-off.


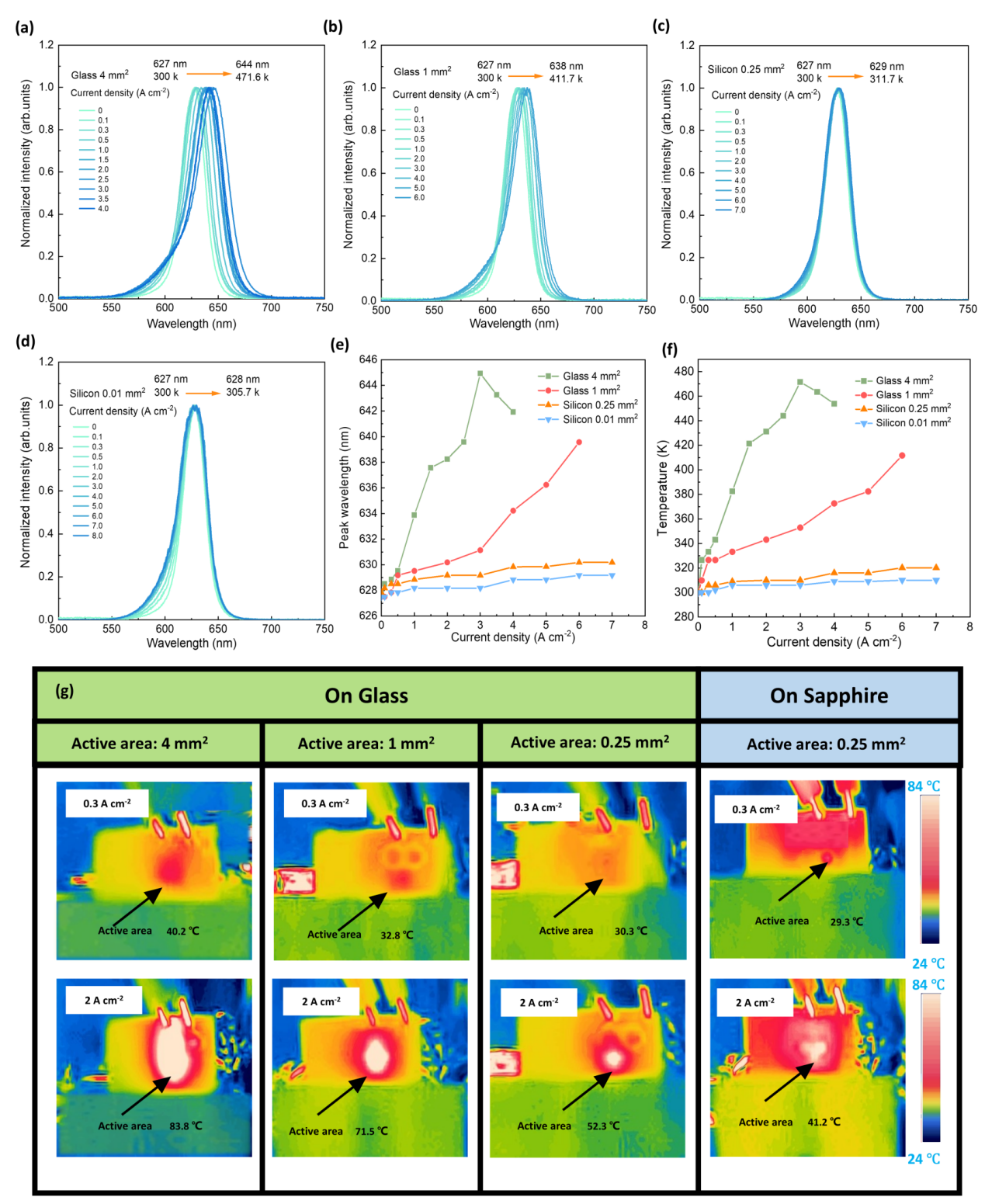


**Supplementary Figure 7. Comparison of spectral redshift and temperature of devices with different areas.** Device structures corresponding to (a), (b) are: Glass/ITO (70 nm)/PEDOT:PSS (45 nm)/TFB (30 nm)/RQD (30 nm)/ZMO (65 nm)/Al, and (c), (d) are: Silicon/Ag (100 nm)/IZO (5 nm)/PEDOT:PSS (45 nm)/TFB (30 nm)/RQD (30 nm)/ZMO (65 nm)/Al (2 nm)/IZO (50 nm). (a)-(d) Comparison of spectral redshift of devices with different areas at different current densities. (e) Summary of the peak wavelength of devices with different areas. (f) The calculated temperature change of devices with different areas. The detail of calculation is shown in Supplementary Note 3. (g) Infrared photographs at the same current of devices with different areas and substrates. Glass or Sapphire/ITO (70 nm)/PEDOT:PSS (45 nm)/TFB (30 nm)/RQD (30 nm)/ZMO (65 nm)/Al.


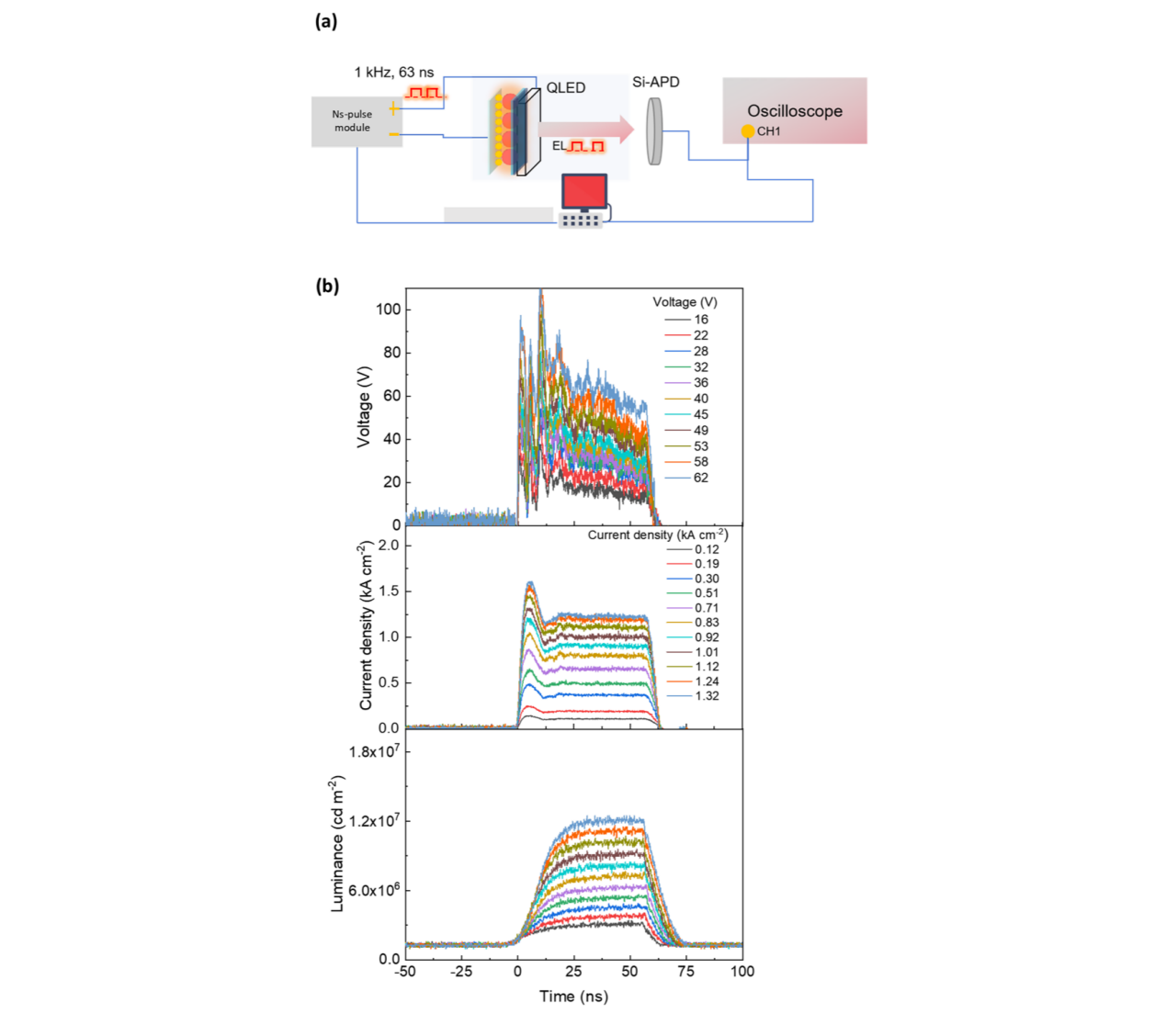


**Supplementary Figure 8. Electrical pulse driving and measurement results.** (a) Schematic diagram of the electrical pulse driving and measurement setup. A custom circuit capable of generating pulsed electrical signals (1 KHz, 63 ns) was connected to the electrodes to drive the devices. Simultaneously, a Si-APD and an oscilloscope are used to detect the pulsed optical signals generated by the devices. (b) The measured voltage, current density and luminance of a single pulse.


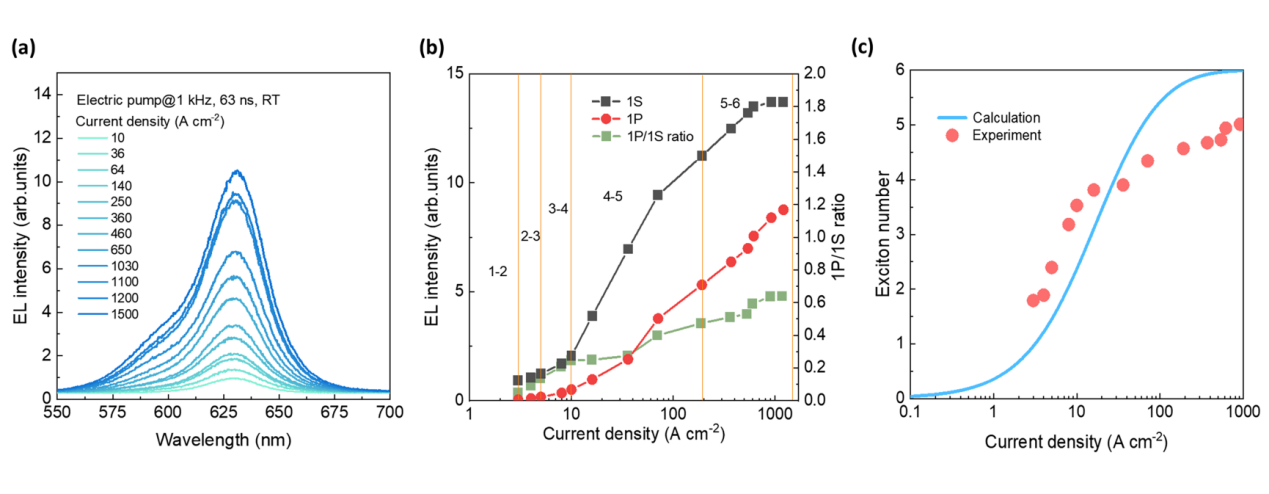


**Supplementary Figure 9. The average QD occupancy calculated by the ratio of 1P/1S EL intensity.** (a) The EL spectra recorded from BE-QLED with different current density. (b) The dependence of the average QD occupancy <*N*>_EL_ on electrical pumping calculated from 1P/1S EL intensity. (c) The average QD occupancy <*N*>_EL_ from the measured EL compare with the calculation. The exciton number are computed based on Supplementary Note 2 and Supplementary Note 4.


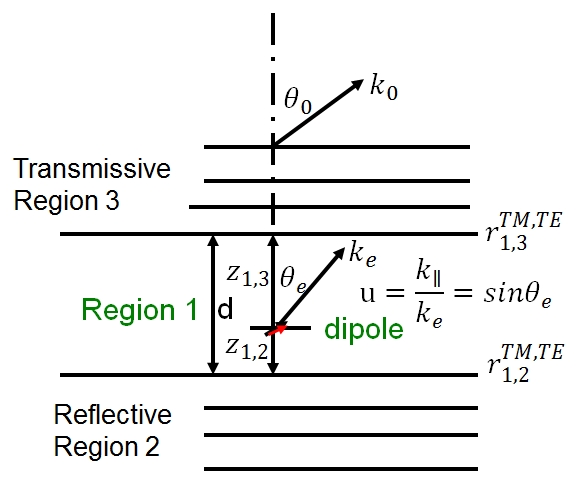


**Supplementary Figure 10.** Optical model of a QLED device.


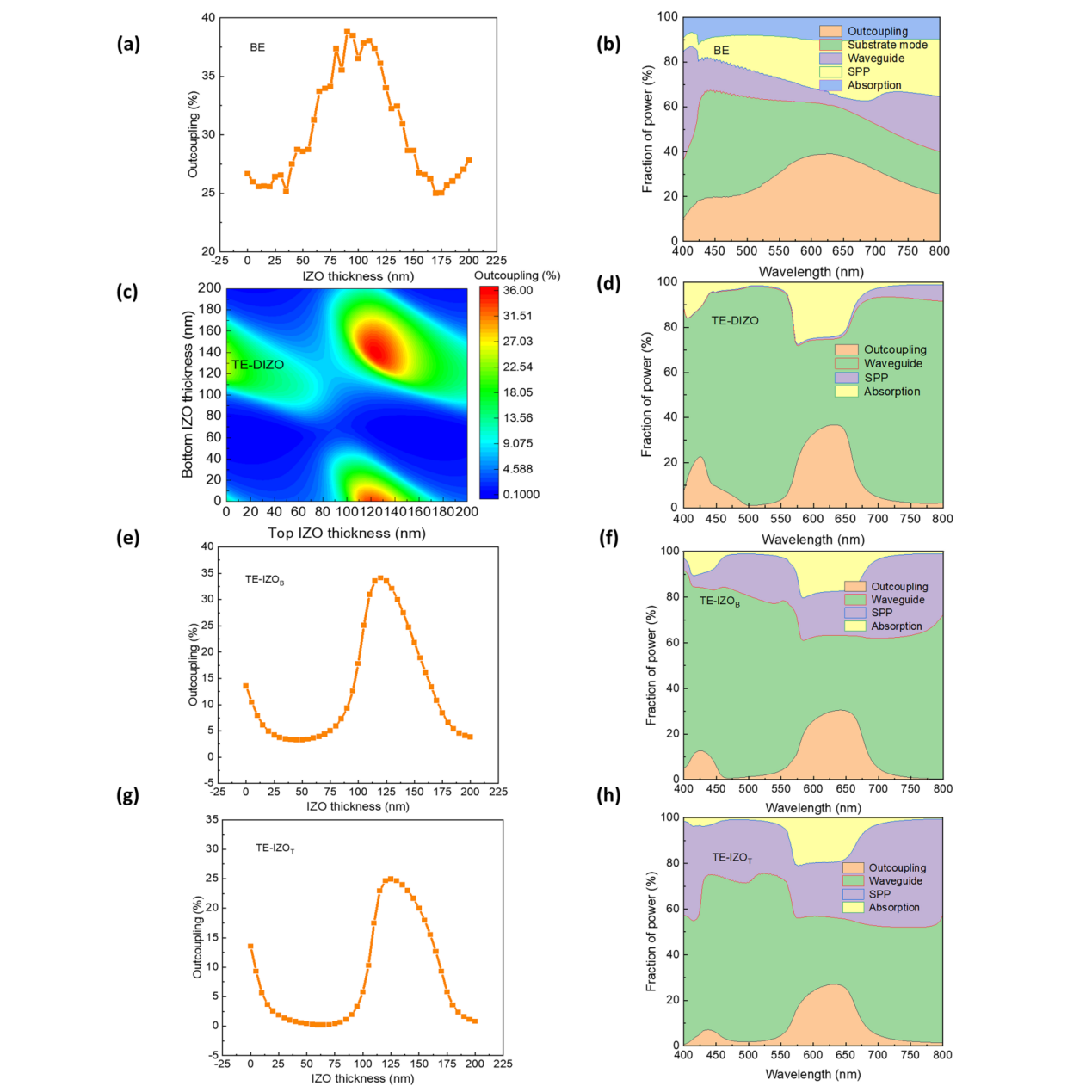


**Supplementary Figure 11. Optical modeling results of QLEDs with different structures.** (a)-(b) Outcoupling and fraction of power of BE (c)-(d) Outcoupling and fraction of power of TE-DIZO (e)-(f) Outcoupling and fraction of power of TE-IZO_B_ (g)-(h) Outcoupling and fraction of power of TE-IZO_T_.


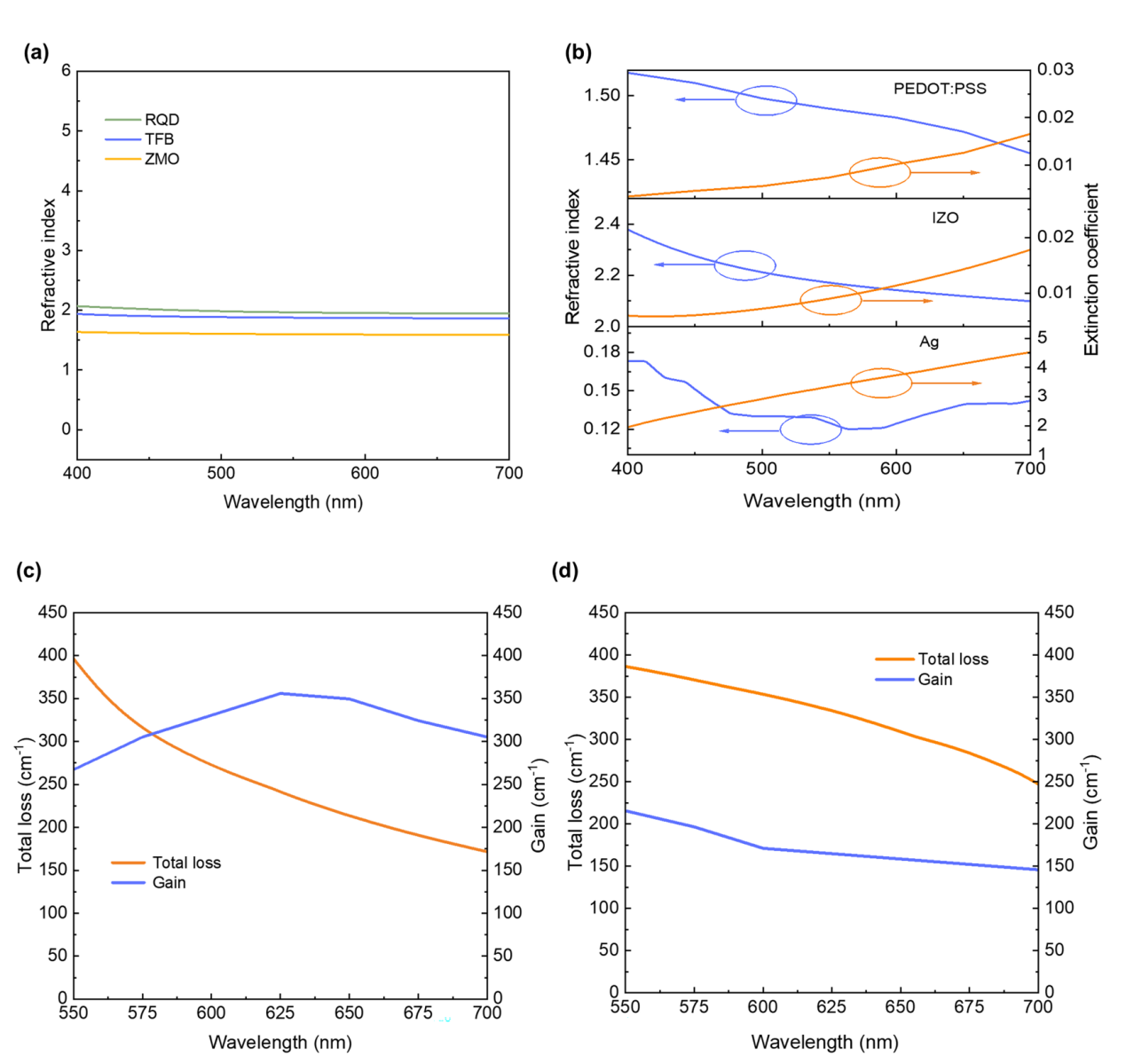


**Supplementary Figure 12. Optical modeling of gain and loss coefficients.** (a) and (b) The refractive indexes and extinction coefficient of materials used in experiment. (c) Total loss and gain of TE_0_ mode of BE structure. (d) Total loss and gain of TE_0_ mode of TE-DIZO structure.


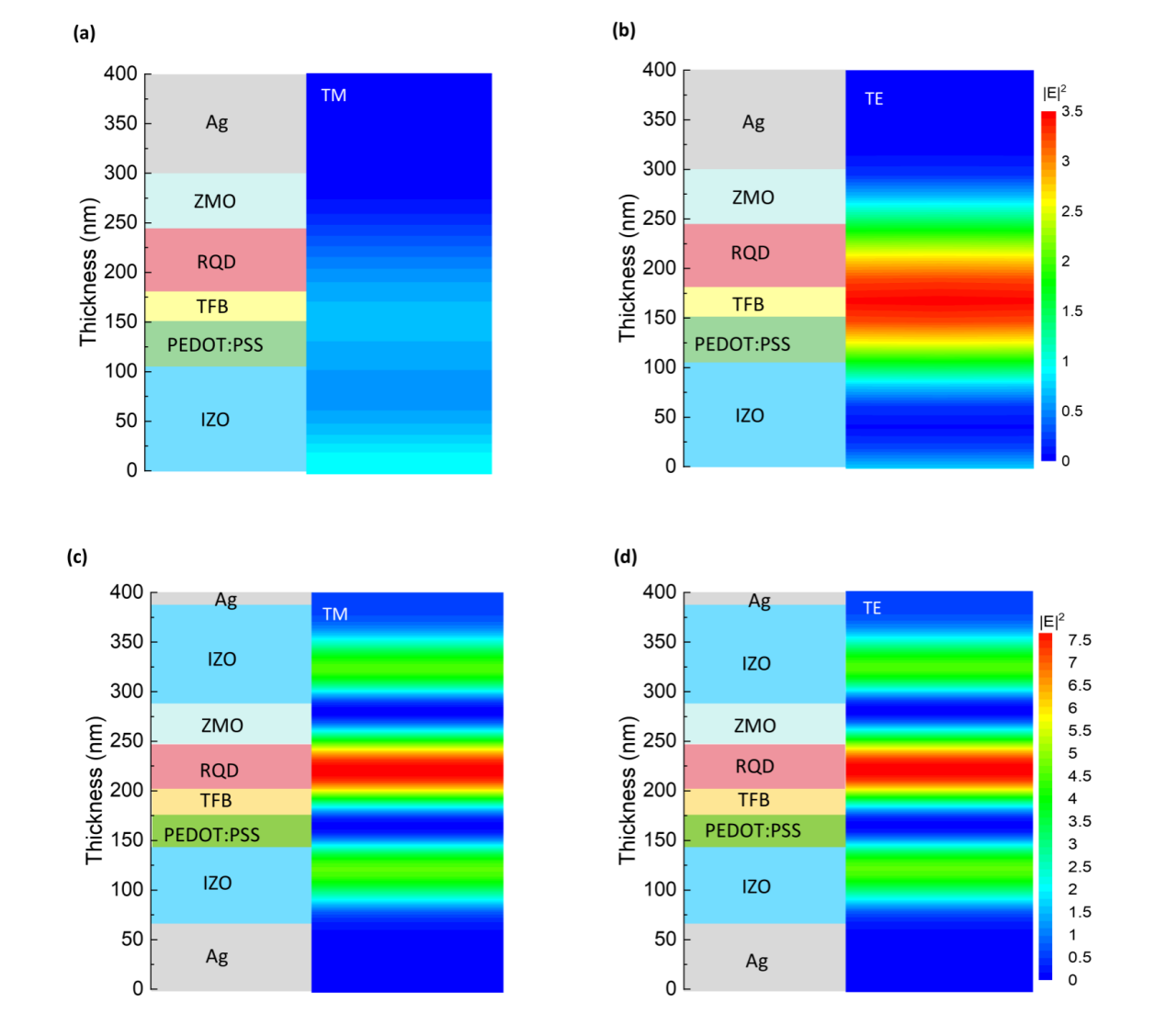


**Supplementary Figure 13. Simulations of transverse magnetic (TM_0_) and transverse electric (TE_0_) modes for BE and TE-DIZO device.** (a), (b) TM_0_ and TE_0_ modes for BE device. The presence of the metal anode, leads to strong quenching of TM_0_ modes. (c), (d) TM_0_ and TE_0_ modes for TE-DIZO device. Due to the presence of thick IZO layers, the quenching of TM_0_ modes is mitigated, resulting in strong localization for both TM_0_ and TE_0_ modes.


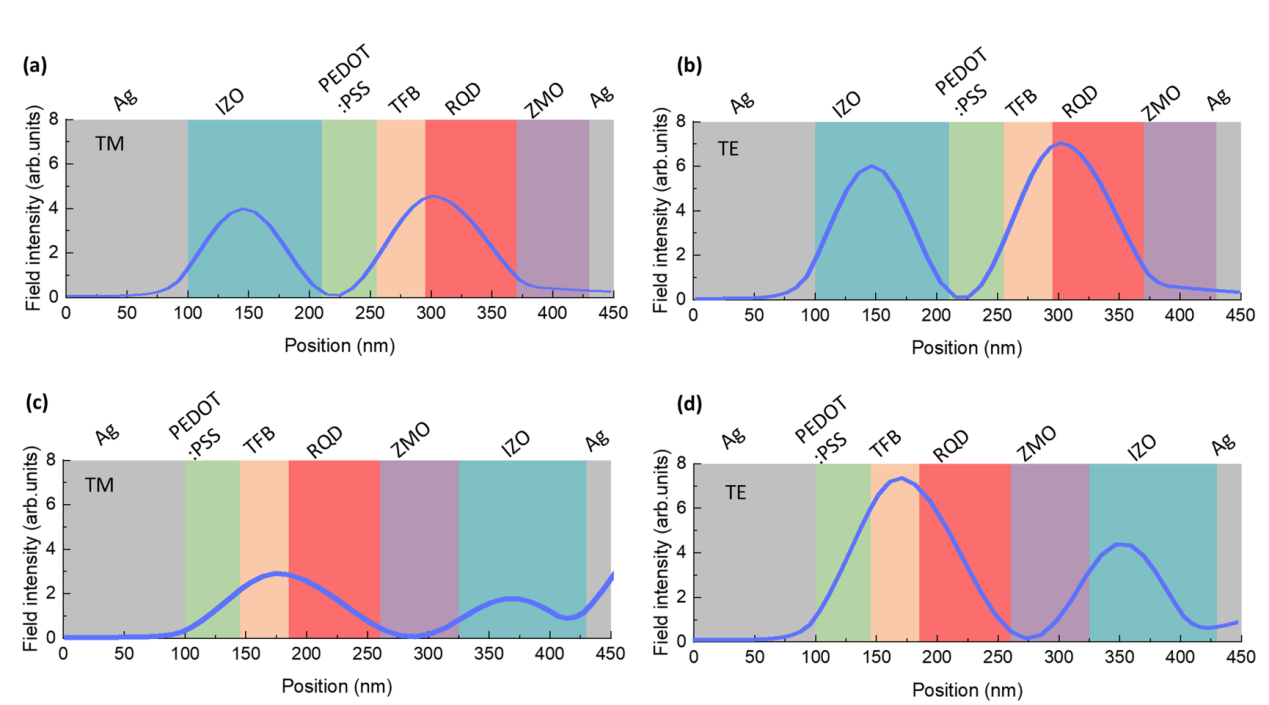


**Supplementary Figure 14. Finite-difference time-domain (FDTD) simulations of TM_0_ and TE_0_ mode propagation along the TE-IZO_B_ and TE-IZO_T_ device.** FDTD simulations of (a) TM_0_ and (b) TE_0_ mode propagation along the TE-IZO_B_ device. FDTD simulations of (c) TM_0_ and (d) TE_0_ mode propagation along the TE-IZO_T_ device.


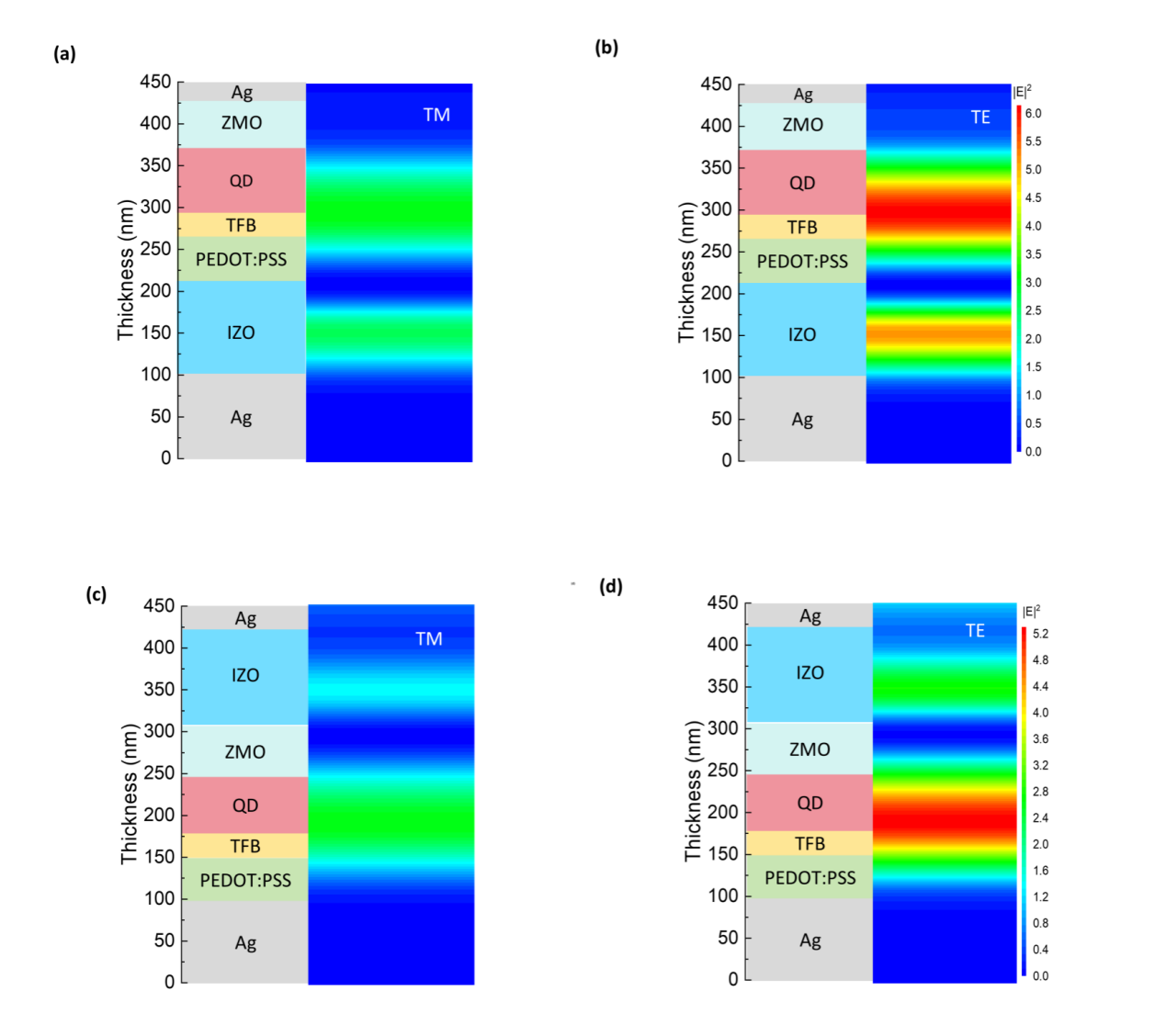


**Supplementary Figure 15. Simulations of TM_0_ and TE_0_ modes for TE-IZO_B_ and TE-IZO_T_ device.** (a), (b) TM_0_ and TE_0_ modes for TE-IZO_B_ device. (c), (d) TM_0_ and TE_0_ modes for TE-IZO_T_ device.


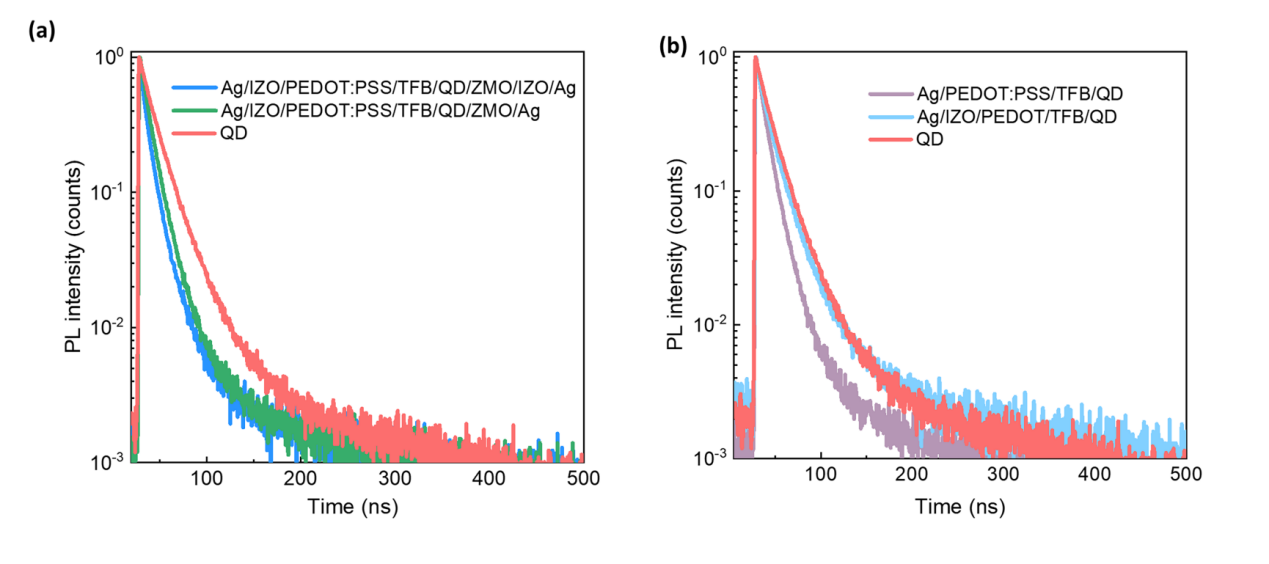


**Supplementary Figure 16. The TRPL decay characteristics.** (a) The PL dynamic of QD, TE-IZO_B_ and TE-DIZO. The reduction of lifetime is due to the accelerated radiative recombination caused by microcavity effects. (b) The PL dynamic of QD in different structure demonstrate the insert of IZO between Ag and QD layer can eliminated the exciton quenching induced by Ag. The addition of IZO layers significantly reduces exciton quenching induced by Ag electrodes.


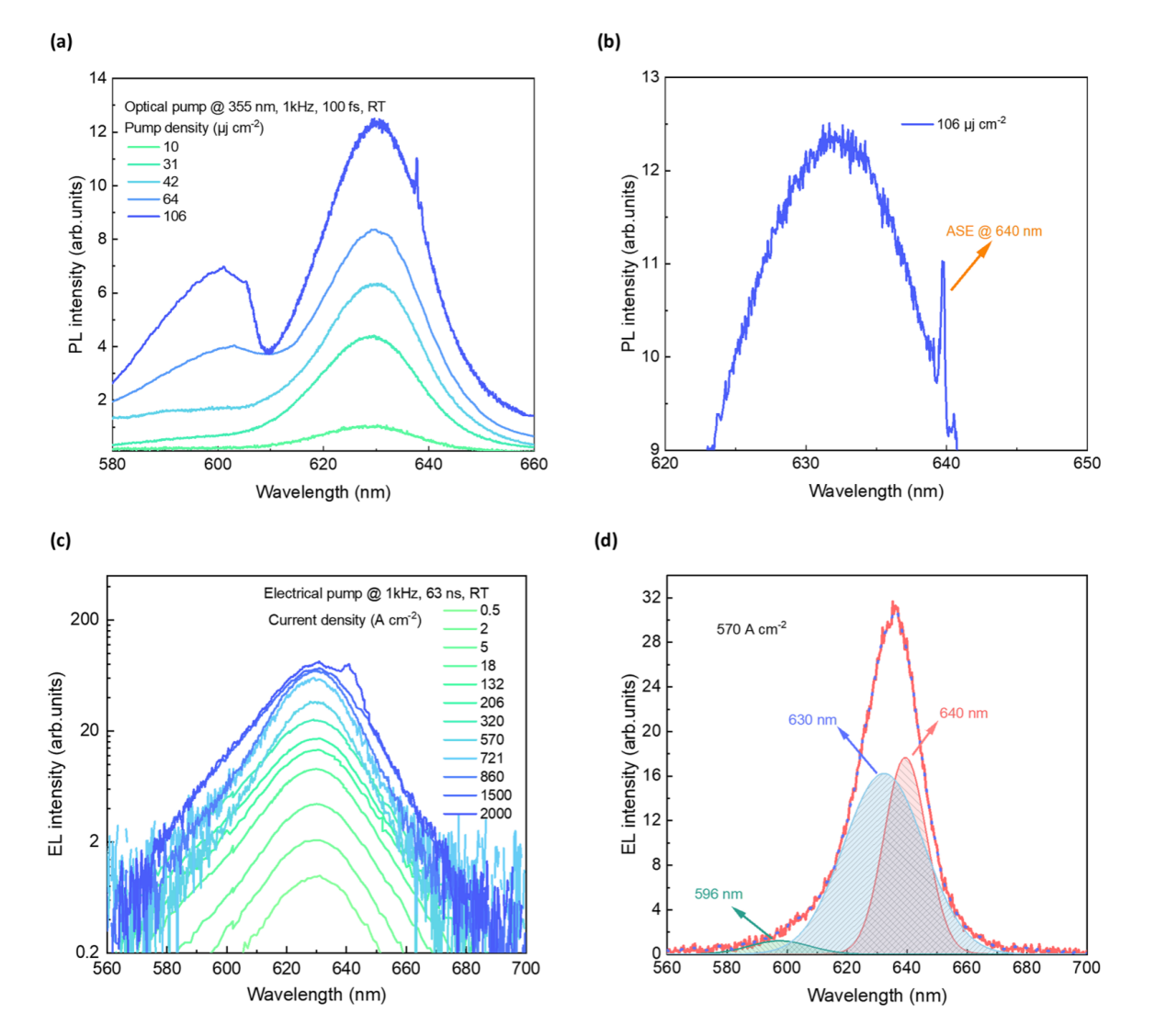


**Supplementary Figure 17. The spectra of TE-DIZO at RT.** (a), (b) The PL spectra of TE-DIZO at RT. (c), (d) The EL spectra of TE-DIZO at RT. Both PL and EL spectra exhibit weak ASE peaks. The EL spectra can be deconvolved into three Lorentzian bands when the current density is 570 A cm^-2^.


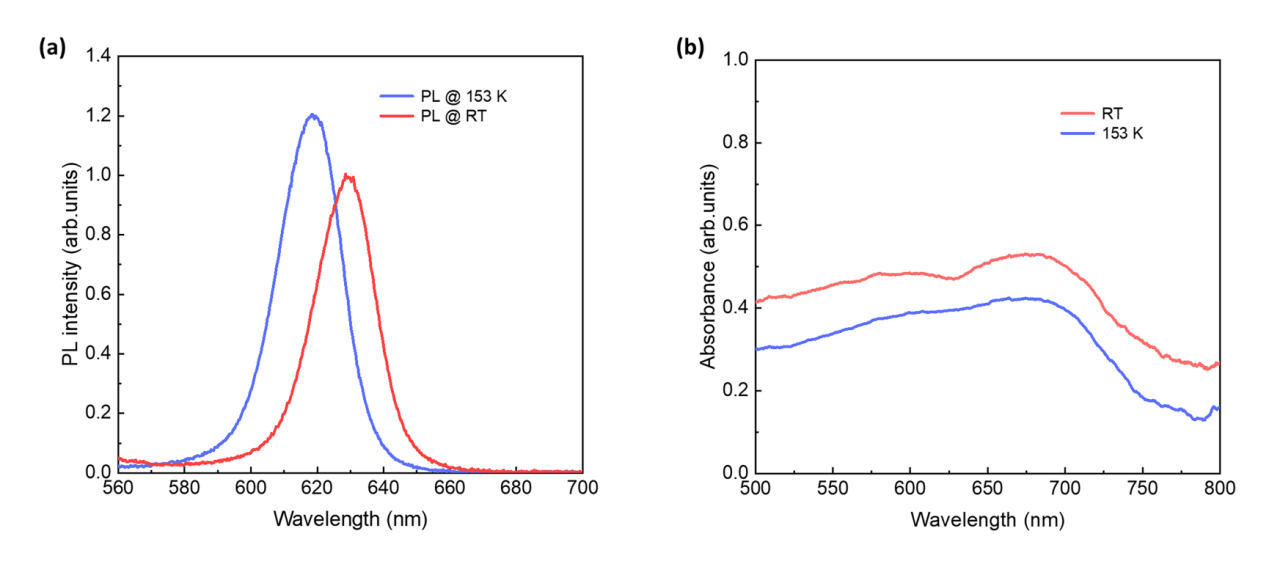


**Supplementary Figure 18. The comparison of PL spectra and absorbance at RT and 153 K of TE-DIZO device.** (a) Comparison of PL spectra at RT and 153 K. (b) Comparison of absorbance at RT and 153 K. The results indicate that low temperatures can reduce device losses.


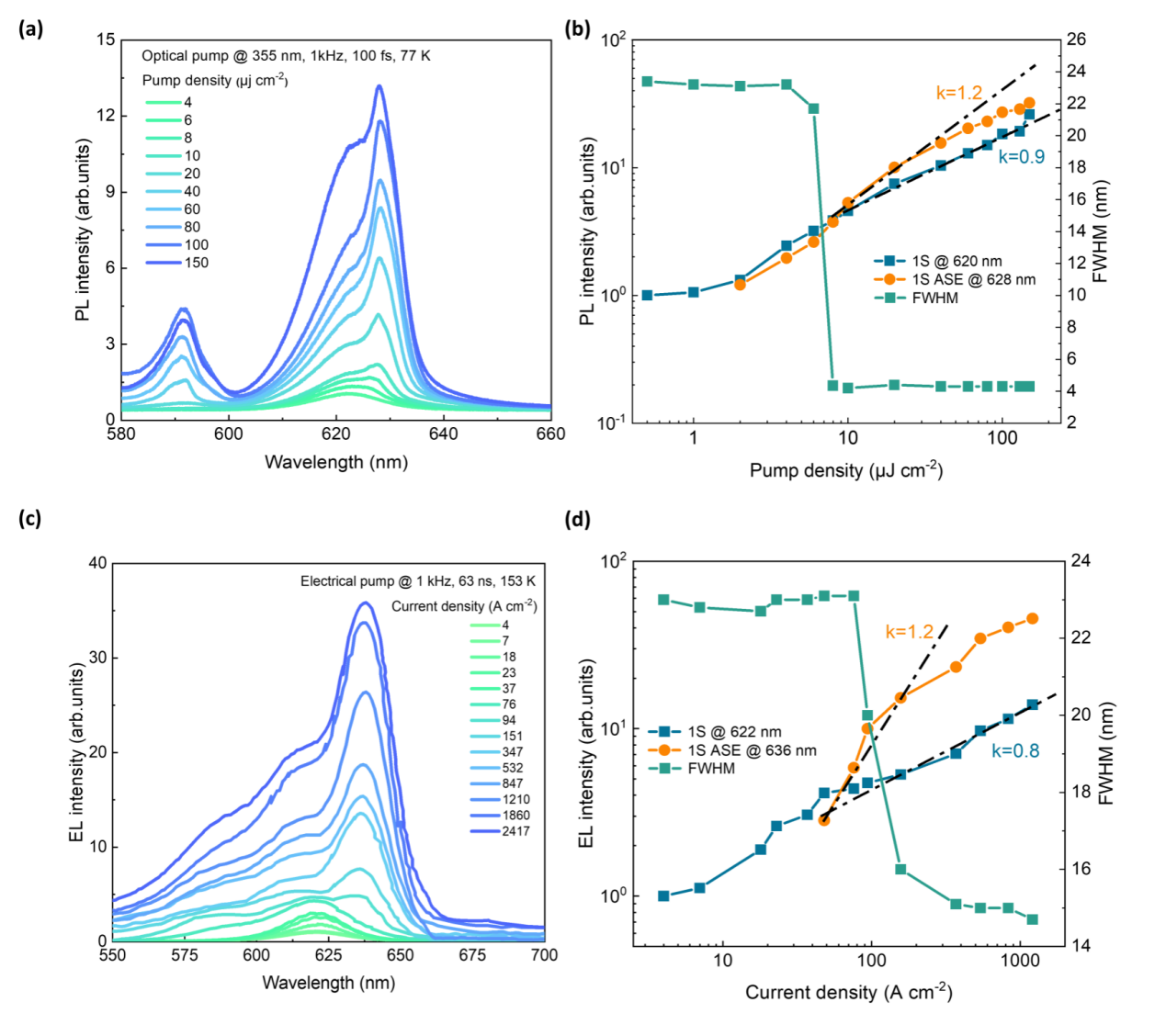


**Supplementary Figure 19. The spectra of TE-DIZO at low temperature.** (a) The PL spectra of TE-DIZO at 77 K. (b) The dependence of the PL intensity and FWHM on pump density. (c) The EL spectra of TE-DIZO at 153 K. (d) The dependence of the EL intensity and FWHM on current density.


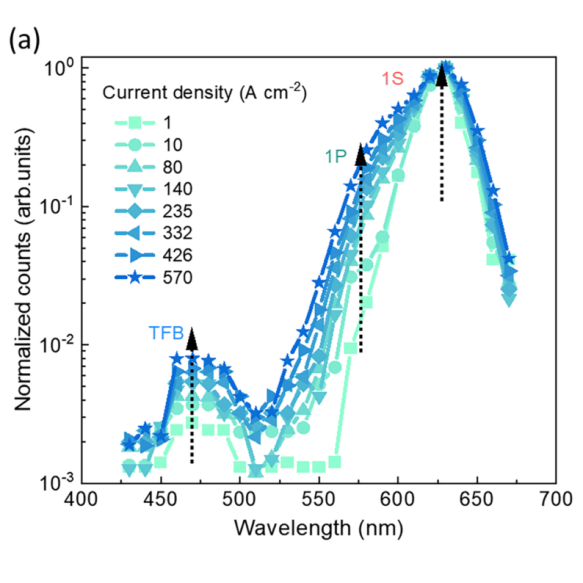


**Supplementary Figure 20**. (a) The EL spectra obtained by single photon counting (SPC) technique, which is integrated with an Edinburgh FS5 system. By measuring the number of photons at different wavelength, we are able to plot the full spectra of the devices. The peak at 470 nm wavelength represents the luminescence of TFB caused by electron leakage.


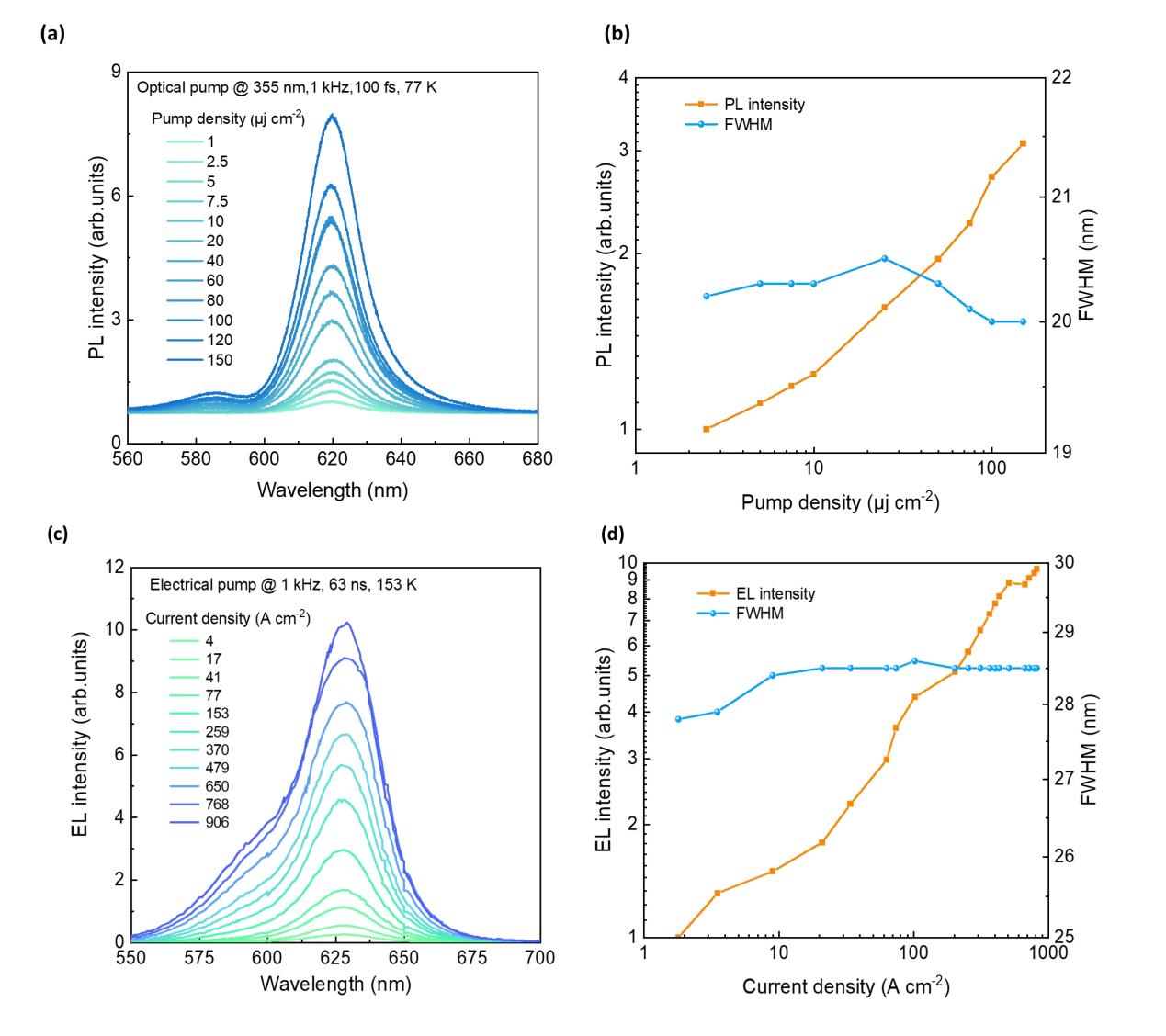


**Supplementary Figure 21. The spectra of BE at low temperature.** (a) The PL spectra of BE at 77 K. (b) The dependence of the PL intensity and FWHM on pump density. (c) The EL spectra of BE at 153 K. (d) The dependence of the EL intensity and FWHM on current density. The results show that no ASE effect was observed.


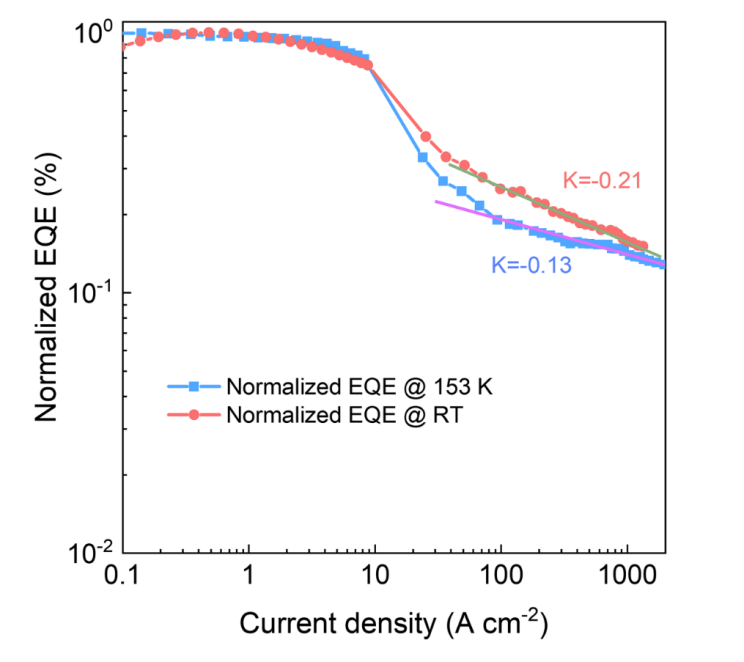


**Supplementary Figure 22.** The comparison of the normalized EQE at room temperature and at 153k, the EQE roll-off at 153k is significantly smaller.

**Supplementary Table 1.** **The lifetime of multiexciton.** Total (*τ*), radiative (*τ_r_*), Auger (*τ_a_*) lifetimes and total (*γ_r_*) emission rates of the 2-6 exciton states in QDs was computed based on the measured single-exciton and biexciton lifetimes. The single exciton lifetime was computed from Supplementary Fig. 2(a) red line. According to equation (7)-(9), we can get 1-6 excitons lifetime ^[2, 3]^.

|  | *τ* (ns) | *τ_r_* (ns) | *τ_a_* (ns) | *γ* |
| --- | --- | --- | --- | --- |
| x | 10 | 10 | 0 | 100% |
| 2x | 0.35 | 2.5 | 0.32 | 13.8% |
| 3x | 0.12 | 0.15 | 0.067 |  |
| 4x | 0.070 | 0.039 | 0.025 |  |
| 5x | 0.045 | 0.016 | 0.012 |  |
| 6x | 0.032 | 0.0085 | 0.0067 |  |

**Supplementary Table 2. The summary of TRPL lifetime**

|  | QD | Ag/PEDOT:PSS/TFB/QD | Ag/IZO/PEDOT:PSS/TFB/QD | Ag/IZO/PEDOT:PSS/TFB/QD/ZMO/Ag | Ag/IZO/PEDOT:PSS/TFB/QD/ZMO/IZO/Ag |
| --- | --- | --- | --- | --- | --- |
| *τ_1_* | 11.56 ns | 8.41 ns | 10.97 ns | 8.44 ns | 5.59 ns |
| *A_1_* | 62.54% | 58.82% | 54.77% | 57.36% | 49.95% |
| *τ_2_* | 30.75 ns | 24.78 ns | 28.77 | 25.29 ns | 22.07 ns |
| *A_2_* | 37.46% | 41.18% | 45.23% | 42.64% | 50.05% |
| *τ_av_* | 23.35 ns | 19.43 ns | 23.15 ns | 20.07 ns | 18.74 ns |

**References**

1. Lim, J., Park, Y. S. & Klimov, V. I. Optical gain in colloidal quantum dots achieved with direct-current electrical pumping. *Nature Materials* **17**, 42-49 (2018).
2. Klimov, V. I. Multicarrier interactions in semiconductor nanocrystals in relation to the phenomena of auger recombination and carrier multiplication. *Annu. Rev. Condens. Annual Review of Condensed Matter Physics* **5**, 285-316 (2014).
3. Klimov, V. I. et al. Scaling of multiexciton lifetimes in semiconductor nanocrystals. *Physical Review B* **77**, 195324 (2008).
4. Jung, H. et al. Two-band optical gain and ultrabright electroluminescence from colloidal quantum dots at 1000 A cm^−2^. *Nature Communications* **13**, 3734 (2022).
5. Sun, Y. et al. Investigation on thermally induced efficiency roll-off: toward efficient and ultrabright quantum-dot light-emitting diodes. *ACS Nano* **13**, 11433–11442 (2019).
6. Neyts K. A. Simulation of light emission from thin-film microcavities. *Journal of The Optical Society of America A-optics Image Science and Vision* **15**, 962–971, (1998).
7. Barnes W.L. Electromagnetic Crystals for Surface Plasmon Polaritons and the Extraction of Light from Emissive Devices. *Journal of Lightwave Technology* **17**, 2170–2182, (1999).
8. Furno, M. et al. Efficiency and rate of spontaneous emission in organic electroluminescent devices. *Physical Review B* **85**, 115205, (2012).
9. Barnes, W. L. Fluorescence near interfaces: The role of photonic mode density. *Journal Of Modern Optics* **45**, 661–699, (1998).
